# Comparative Genomics Reveals Ecological and Evolutionary Insights into Sponge-Associated *Thaumarchaeota*

**DOI:** 10.1128/mSystems.00288-19

**Published:** 2019-08-13

**Authors:** Shan Zhang, Weizhi Song, Bernd Wemheuer, Julie Reveillaud, Nicole Webster, Torsten Thomas

**Affiliations:** a School of Biotechnology and Biomolecular Sciences, University of New South Wales, Sydney, Australia; b Center for Marine Science & Innovation, University of New South Wales, Sydney, Australia; c School of Biological, Earth and Environmental Sciences, University of New South Wales, Sydney, Australia; d ASTRE, INRA, CIRAD, University of Montpellier, Montpellier, France; e Australian Institute of Marine Science, Townsville, Australia; f Australian Centre for Ecogenomics, The University of Queensland, Brisbane, Australia; University of Vienna

**Keywords:** evolution, genetic features, host associated, sponge microbiome, symbionts

## Abstract

*Thaumarchaeota* are frequently reported to associate with marine sponges (phylum Porifera); however, little is known about the features that distinguish them from their free-living thaumarchaeal counterparts. In this study, thaumarchaeal metagenome-assembled genomes (MAGs) were reconstructed from metagenomic data sets derived from the marine sponges Hexadella detritifera, *Hexadella* cf. *detritifera*, and Stylissa flabelliformis. Phylogenetic and taxonomic analyses revealed that the three thaumarchaeal MAGs represent two new species within the genus *Nitrosopumilus* and one novel genus, for which we propose the names “*Candidatus* ^U^Nitrosopumilus hexadellus,” “*Candidatus* ^U^Nitrosopumilus detritiferus,” and “*Candidatus* ^U^Cenporiarchaeum stylissum” (the U superscript indicates that the taxon is uncultured). Comparison of these genomes to data from the Sponge Earth Microbiome Project revealed that “*Ca*. ^U^Cenporiarchaeum stylissum” has been exclusively detected in sponges and can hence be classified as a specialist, while “*Ca*. ^U^Nitrosopumilus detritiferus” and “*Ca*. ^U^Nitrosopumilus hexadellus” are also detected outside the sponge holobiont and likely lead a generalist lifestyle. Comparison of the sponge-associated MAGs to genomes of free-living *Thaumarchaeota* revealed signatures that indicate functional features of a sponge-associated lifestyle, and these features were related to nutrient transport and metabolism, restriction-modification, defense mechanisms, and host interactions. Each species exhibited distinct functional traits, suggesting that they have reached different stages of evolutionary adaptation and/or occupy distinct ecological niches within their sponge hosts. Our study therefore offers new evolutionary and ecological insights into the symbiosis between sponges and their thaumarchaeal symbionts.

**IMPORTANCE** Sponges represent ecologically important models to understand the evolution of symbiotic interactions of metazoans with microbial symbionts. *Thaumarchaeota* are commonly found in sponges, but their potential adaptations to a host-associated lifestyle are largely unknown. Here, we present three novel sponge-associated thaumarchaeal species and compare their genomic and predicted functional features with those of closely related free-living counterparts. We found different degrees of specialization of these thaumarchaeal species to the sponge environment that is reflected in their host distribution and their predicted molecular and metabolic properties. Our results indicate that *Thaumarchaeota* may have reached different stages of evolutionary adaptation in their symbiosis with sponges.

## Abstract (precis)

Sponges represent ecologically important models to understand the evolution of symbiotic interactions of metazoans with microbial symbionts. *Thaumarchaeota* are commonly found in sponges, but their potential adaptations to a host-associated lifestyle are largely unknown. Here, we present three novel sponge-associated thaumarchaeal species and compare their genomic and predicted functional features with those of closely related free-living counterparts. We found different degrees of specialization of these thaumarchaeal species to the sponge environment that is reflected in their host distribution and their predicted molecular and metabolic properties. Our results indicate that *Thaumarchaeota* may have reached different stages of evolutionary adaptation in their symbiosis with sponges.

## INTRODUCTION

Marine sponges are a group of sessile animals, which harbor diverse microorganisms that often form stable and specific associations with their host (1–3). Phylogenetic analyses have shown that sponge microbiota often contain members of the phyla *Proteobacteria* (mainly *Gamma*- and *Alphaproteobacteria*), *Actinobacteria*, *Firmicutes*, *Chloroflexi*, *Nitrospirae*, *Cyanobacteria*, “*Candidatus* Poribacteria,” and *Thaumarchaeota* (4–6). Studies analyzing isolate genomes or metagenome-assembled genomes (MAGs) from a few of these bacterial phyla have postulated several adaptive features of sponge-associated symbionts compared to their free-living relatives, including an enrichment of unique eukaryotic-like proteins (ELPs) and methyltransferases in the cyanobacterial “*Candidatus* Synechococcus spongiarum” (7–9), an abundance of diverse phyH domain proteins in Poribacteria (10), an enrichment of CRISPR-Cas systems, ABC transporters, and restriction-modification systems in the alphaproteobacterial *Rhodospirillaceae* (11), and functions related to carbohydrate uptake, phage defense, and protein secretion in sulfur-oxidizing *Gammaproteobacteria* (12, 13). In contrast, other *Proteobacteria* such as the genera *Aquimarina* and *Pseudovibrio* have few or no obvious features that distinguish them from their free-living counterparts (9).

*Thaumarchaeota* occur in diverse habitats, including seawater (14–17), hot springs (18), freshwater (19), industrial wastewater (20, 21), terrestrial soil (22–25), marine sediments (26, 27), and marine sponges (28–33). Previous work has shown that some *Thaumarchaeota* form so-called “sponge-specific” or “sponge-enriched” clades, which are defined by monophyletic 16S rRNA sequence clusters found either exclusively or highly enriched in sponges compared to other environments (1, 5, 34, 35). This indicates that some *Thaumarchaeota* clades might have diverged from their free-living counterparts because of adaptation to a sponge-associated lifestyle. *Thaumarchaeota* are known to be ecologically important autotrophic, aerobic ammonia oxidizers, which use urea and probably creatinine as indirect sources of ammonia (23, 36) and produce NO (37), N_2_O (38), and ether-lipid components (39). A possible uptake of urea and amino acids and the expression of a CO_2_ fixation pathway have also been recently reported for a sponge-associated *Thaumarchaeota* (33), but no other genomic features indicative of adaptation to the sponge environment have been reported.

This study aimed to generate hypotheses on the potential functional adaptations based on the comparative genomic analyses of novel thaumarchaeal symbionts of sponges. For this purpose, we reconstructed MAGs from metagenomic data derived from the sponges *Stylissa flabelliformis*, *Hexadella detritifera*, and *Hexadella* cf. *detritifera* and compared them to genomes from closely related free-living *Thaumarchaeota*.

## RESULTS AND DISCUSSION

### MAG reconstruction and phylogenetic analyses define novel thaumarchaeal taxa.

The seven sponge specimens investigated in this study (Table 1) each contained a single 16S rRNA gene sequence belonging to the phylum *Thaumarchaeota* as reconstructed using the Mapping-Assisted Targeted Assembly for Metagenomics (MATAM) algorithm (40). Similarly, only one thaumarchaeal MAG was recovered for each sample, to which the 16S rRNA gene sequence could be aligned with high similarity (99.95% ± 0.08%). MAGs had average sizes of 1.18 ± 0.08 Mb, no heterogeneity, contamination levels of 0.57% ± 0.51% and completeness of 84.36% ± 10.91% as assessed by CheckM (41).

**TABLE 1:** Statistics of sponge-associated thaumarchaeal MAGs

Sample	MAG	Proposed taxon^*a*^	Host species	MAG size (Mbp)	Completeness (%)	Estimated genome size (Mbp)	Contamination	Avg coverage of MAG	16S rRNA gene length (bp)
B06	HdNhB06	“*Ca*. ^U^N. hexadellus”	*Hexadella dedritifera*	1.25	91.83	1.36	0.96	28×	1,365
D6	HdNhD6	“*Ca*. ^U^N. hexadellus”	*Hexadella dedritifera*	1.12	92.31	1.21	0	89×	1,041
H08	HdNdH8	“*Ca*. ^U^N. detritiferus”	*Hexadella* cf. *dedritifera*	1.13	89.26	1.27	0	11×	1,448
H13	HdNdH13	“*Ca*. ^U^N. detritiferus”	*Hexadella* cf. *dedritifera*	1.12	89.9	1.25	0.07	106×	1,600
S13	SfCsS13	“*Ca*. ^U^C. stylissum”	*Stylissa flabelliformis*	1.31	85.58	1.53	0.96	9×	1,432
S14	SfCsS14	“*Ca*. ^U^C. stylissum”	*Stylissa flabelliformis*	1.13	80.13	1.41	1.04	6×	1,430
S15	SfCsS15	“*Ca*. ^U^C. stylissum”	*Stylissa flabelliformis*	1.17	61.54	1.90	0.96	5×	1,431

a The three proposed taxa are “*Candidatus* ^U^Nitrosopumilus hexadellus,” “*Candidatus* ^U^Nitrosopumilus detritiferus,” and “*Candidatus* ^U^Cenporiarchaeum stylissum.”

**FIG 1:**
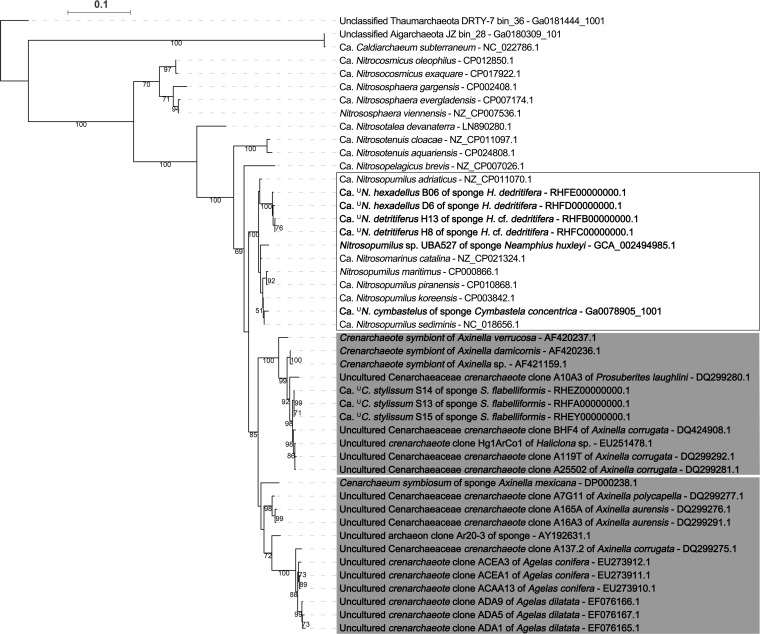
Maximum-likelihood tree based on 16S rRNA gene sequences (≥1,000 bp) for sponge-associated and free-living *Thaumarchaeota*. Results ≥50% are shown for 1,000 bootstraps. The tree is rooted with two Aigarchaeota (“*Candidatus* Caldiarchaeum subterraneum” and an unclassified Aigarchaeota) and one unclassified *Thaumarchaeota*, and sponge-derived sequences are displayed in boldface type. Gray shaded boxes represent sponge-speciﬁc monophyletic clusters, and white boxes represent monophyletic clusters containing both sponge-derived sequences and free-living sequences. Bars indicate 10% sequence divergence.

Maximum-likelihood trees were constructed for the 16S rRNA genes or single-copy genes (SCG) from the MAGs and genomes of their close relatives in the National Center for Biotechnology Information (NCBI) nonredundant nucleotide (NT) database (see Table S1 in the supplemental material). These two trees showed very similar patterns, with the sponge-derived *Thaumarchaeota* falling into two distinct clades (Fig. 1; see also Fig. S1 in the supplemental material). One clade was comprised of the three MAGs from *Stylissa flabelliformis*, which were 99.91% ± 0.04% similar to each other at the 16S rRNA gene level and formed an apparent sister clade (98.81% ± 0.11% similar) to “Candidatus Cenarchaeum symbiosum” (GenBank accession number DP000238.1), which was previously reported from the marine sponge genus *Axinella* (30). The other clade was composed of four genomes from the *Hexadella* samples, which were related to the free-living Nitrosopumilus maritimus (GenBank accession number CP000866.1) from seawater. The reconstructed 16S rRNA gene sequences of this clade were 98.65% ± 0.31% similar to other sequences from the genus *Nitrosopumilus* and had 99.58% ± 0.20% similarity to each other. Our analysis also included two MAGs (GCA_002494985.1 and GCA_002506665.1) from the Genome Taxonomy Database (GTDB), one of which (GCA_002494985.1) also contained a partial 16S rRNA gene. These MAGs were derived from a sample of the deep-sea sponge Neamphius huxleyi. However, in the phylogenetic trees, these organisms clustered separately from each other as well as from the MAGs analyzed here and were more closely related to free-living *Thaumarchaeota*. They were therefore not further analyzed.

10.1128/mSystems.00288-19.1 FIG S1 Maximum-likelihood trees of 16S rRNA gene sequences (A) and SCG (B) for sponge-derived *Thaumarchaeota* and free-living relatives. Results of ≥50% are shown for 1,000 bootstraps. The trees are rooted with two Aigarchaeota (“*Candidatus* Caldiarchaeum subterraneum” and an unclassified Aigarchaeota) and one unclassified *Thaumarchaeota*, and sponge-derived sequences are displayed in boldface type. Shaded boxes represent sponge-speciﬁc monophyletic clusters, and open boxes represent monophyletic clusters containing both sponge-derived sequences and free-living sequences. Bars indicate 10% sequence divergence. Download FIG S1, TIF file, 2.0 MB. Copyright © 2019 Zhang et al. 2019 Zhang et al. This content is distributed under the terms of the Creative Commons Attribution 4.0 International license.

10.1128/mSystems.00288-19.6 TABLE S1 Details of completed thaumarchaeal reference genomes. Download Table S1, DOCX file, 0.02 MB. Copyright © 2019 Zhang et al. 2019 Zhang et al. This content is distributed under the terms of the Creative Commons Attribution 4.0 International license.

**FIG 2:**
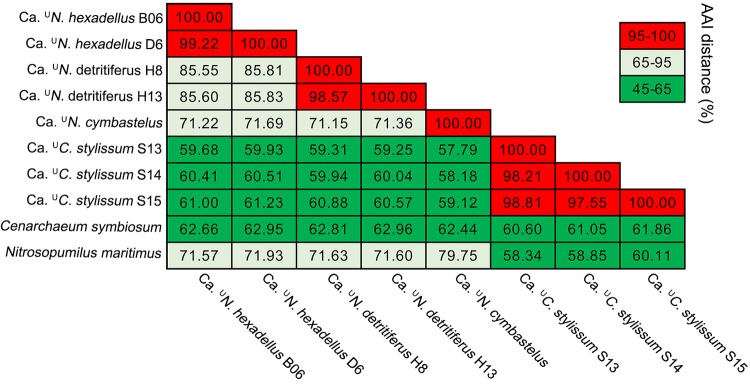
Pairwise average amino acid identity (AAI) distances among the genomes of sponge-derived *Thaumarchaeota* and their closest relatives. The color bar indicates the range of AAI distances that represents different taxonomical levels: the level of species (95 to 100%), genus (65 to 95%), and family (45 to 65%).

The thaumarchaeal MAGs from *Stylissa flabelliformis* had pairwise amino acid identities (AAI) of 57.30% ± 7.30% with “Ca. Cenarchaeum symbiosum” (Fig. 2), suggesting that they represent a different genus within the same family (45 to 65%) based on the criteria proposed by Konstantinidis et al. (42). Pairwise AAI distance between MAGs from *S. flabelliformis* were 98.19% ± 0.63%. We therefore propose the novel genus “*Candidatus* Cenporiarchaeum” with the species “*Candidatus* ^U^Cenporiarchaeum stylissum” (the U superscript indicates that the taxon is uncultured) represented by the MAGs found in *S. flabelliformis*.

MAGs from the *Hexadella* samples had AAI distances of 70.95% ± 14.12% with *Nitrosopumilus* sp. (GCA_000328925.1), indicating that they belong to the same genus (65 to 95%) but are distinct species (42). Pairwise AAI distances between the MAGs from the *Hexadella dedritifera* and *Hexadella* cf. *dedritifera* were 99.22% and 98.57%, respectively. AAI distances between genomes from the two sponge taxa were 85.70% ± 0.14%, indicating two distinct species. We therefore propose the names “*Candidatus* ^U^Nitrosopumilus hexadellus” for the species found in *H. dedritifera*, and “*Candidatus* ^U^Nitrosopumilus detritiferus” for the species found in *H*. cf. *dedritifera*.

Another thaumarchaeal genome (Ga0078905) from the sponge Cymbastela concentrica from a previous study (33) had AAI values of 71.36% ± 0.24% with “*Ca*. ^U^Nitrosopumilus detritiferus” and “*Ca*. ^U^Nitrosopumilus hexadellus,” indicating a different species within the same genus, and we propose here the name “*Candidatus* ^U^Nitrosopumilus cymbastelus.”

### Size and GC content of sponge-associated thaumarchaeal genomes.

Genome reduction is often considered a signature of microbial symbiosis, as genes no longer required for a host-associated lifestyle are being lost (43). Interestingly though, no evidence for genome reduction has been reported for archaea. In our study, we estimated the genome sizes of MAGs based on the predicted degree of genome completeness evaluated with 146 lineage-specific SCG at a rank of phylum using CheckM (41). The “*Ca*. ^U^Cenporiarchaeum stylissum” MAG ST15 MAG was excluded from this and all subsequent analyses, as it had comparatively low genome completeness (Table 1).

**FIG 3:**
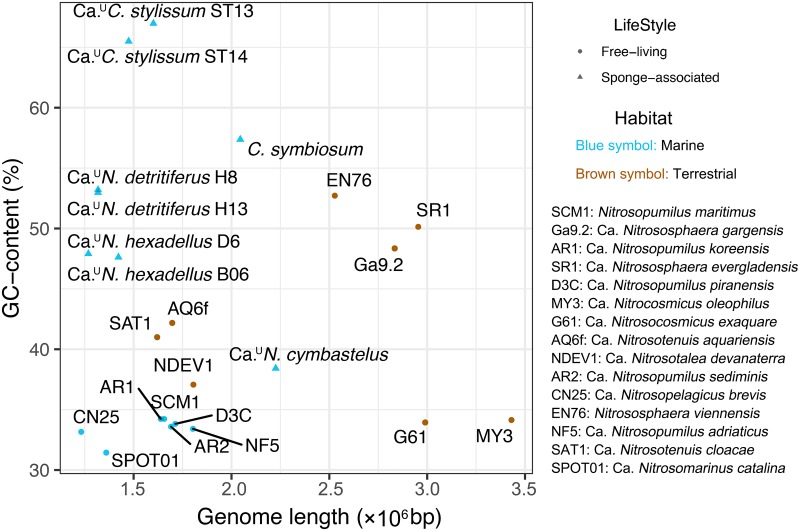
Genome length versus GC content for sponge-derived and free-living *Thaumarchaeota*. Genome length (in base pairs) versus GC content (as a percentage) are shown.

We found that the average estimated genome size of the five sponge-associated thaumarchaeal species (1.51 ± 0.34 Mbp) was significantly smaller than that of 15 terrestrial and marine free-living *Thaumarchaeota* (1.97 ± 0.65 Mbp) (Wilcox test, *P* value = 0.047; Fig. 3), but not when compared only to marine, free-living *Thaumarchaeota* (1.51 ± 0.19 Mbp) (Wilcox test, *P* value = 0.613). The former significant result is however likely due to the fact that marine *Thaumarchaeota* generally have smaller genomes than their terrestrial counterparts (44). Estimated genome sizes of individual sponge-associated species were also not significantly smaller than free-living ones, with “Ca. ^U^Cenporiarchaeum stylissum” and “Ca. ^U^Nitrosopumilus cymbastelus” having the largest of all marine genomes investigated here. This finding is consistent with recent work also noting a lack of significant reduction in genome size for archaeal endosymbionts of ciliates (45, 46).

However, sponge-derived thaumarchaeal species had a significantly higher average GC content (53.75% ± 9.52%) than those of their 15 free-living counterparts (38.22% ± 6.98%) (Wilcox test, *P* value = 7.67E−04) or the 7 free-living marine *Thaumarchaeota* (33.40% ± 0.94%) (Wilcox test, *P* value = 3.11E−04). The GC contents for “*Ca*. ^U^Cenporiarchaeum stylissum” (66.24% ± 1.05%), “*Ca*. ^U^Nitrosopumilus hexadellus” (47.77% ± 0.21%), and “*Ca*. ^U^Nitrosopumilus detritiferus” (53.10% ± 0.16%) were also found separately to be higher than those of the seven free-living marine *Thaumarchaeota*, but with only marginal statistical support (Wilcox test, *P* value = 0.056 for each respective pairwise test). “*Ca*. ^U^Nitrosopumilus cymbastelus” had a GC content closer to those of free-living, marine *Thaumarchaeota*. GC enrichment has previously also been observed in obligate bacterial symbionts, such as “*Candidatus* Hodgkinia cicadicola” and “*Candidatus* Tremblaya princeps” of cicada (insects) (43, 47) and was assumed to be a result of GC directional mutational pressure during genome evolution (47). However, it has also been shown that high GC content is correlated to the adaptation to environmental stresses, such as nutrient and energy limitation (48). The mechanisms that give rise to the high GC content in the sponge-associated symbiotic *Thaumarchaeota* therefore require further investigation.

### Analysis of host specificity showed generalist and specialist taxa.

The thaumarchaeal phylogenetic tree based on 16S rRNA gene sequences from the current study and 19 sponge-derived thaumarchaeal sequences previously defined as sponge-specific sequence clusters by Simister et al. (5) showed that “*Ca*. ^U^Cenporiarchaeum stylissum” and “*Ca*. Cenarchaeum symbiosum” belong to separate monophyletic clades comprised exclusively of sponge-specific 16S rRNA sequences (Fig. 1). This phylogenetic placement indicates that these two organisms might have an obligate sponge-associated lifestyle. In contrast, “*Ca*. ^U^Nitrosopumilus detritiferus” and “*Ca*. ^U^Nitrosopumilus hexadellus” did not cluster with previously described sponge-specific sequences but instead formed a distinct cluster with other free-living *Thaumarchaeota*. “*Ca*. ^U^Nitrosopumilus cymbastelus” was also closely related to sequences from free-living *Thaumarchaeota*.

**FIG 4:**
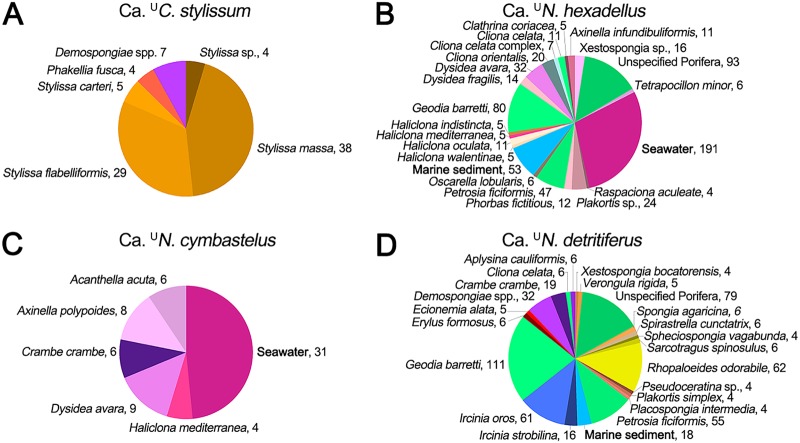
Enrichment of “*Ca*. ^U^Cenporiarchaeum stylissum” (A), “*Ca*. ^U^Nitrosopumilus hexadellus” (B), “*Ca*. ^U^Nitrosopumilus cymbastelus” (C), and “*Ca*. ^U^Nitrosopumilus detritiferus” (D) in samples of the Sponge Earth Microbiome Project. Enrichment was calculated using either presence/absence binomial tests or relative frequency-based rank sum tests. The values following the sample names represent the number of samples in which the archaeal species was detected.

The 16S rRNA genes of “*Ca*. ^U^Cenporiarchaeum stylissum,” “*Ca*. ^U^Nitrosopumilus cymbastelus,” “*Ca*. Cenarchaeum symbiosum,” “*Ca*. ^U^Nitrosopumilus detritiferus,” and “*Ca*. ^U^Nitrosopumilus hexadellus” were further searched against the Sponge Earth Microbiome Project (SEMP) database, which comprises 3,490 samples from more than 250 different sponge species and other marine habitats (http://www.spongeemp.com) (49). We allowed for single mismatches (i.e., 99% similarity) in the search against the zero-distance operational taxonomic units (zOTUs) generated by the Deblur algorithm used in the SEMP (49). “*Ca*. ^U^C. stylissum” was found in 115 samples, which belonged exclusively to six sponge species, including four *Stylissa* species (Fig. 4; Table S2). “*Ca*. ^U^N. hexadellus” and “*Ca*. ^U^N. detritiferus” were detected in 969 and 722 samples, respectively, which belonged mainly to at least 20 sponge species. Both species were also found in some seawater and sediment samples analyzed in the SEMP, but the fact that they were enriched in sponges and had high genome coverage when assembled from the *Hexadella* microbial metagenomes (Table 1) suggests that they are symbionts of sponges.

10.1128/mSystems.00288-19.7 TABLE S2 Enrichment of thaumarchaeal species in different hosts and sample types. Enrichment analysis was calculated by default setting on the online server SEMP. Binomial *P* value test and rank sum (frequency aware) *P* value test were displayed. Download Table S2, DOCX file, 0.02 MB. Copyright © 2019 Zhang et al. 2019 Zhang et al. This content is distributed under the terms of the Creative Commons Attribution 4.0 International license.

“*Ca*. ^U^Nitrosopumilus cymbastelus” was found in 87 samples, nearly half of which were seawater, while the remainder were recovered from five sponge species. Through 16S rRNA gene sequencing and fluorescence *in situ* hybridization visualization, “*Ca*. ^U^N. cymbastelus” has previously been shown to be consistently present in *Cymbastela concentrica*, which was not analyzed in the SEMP (33). “*Ca*. Cenarchaeum symbiosum” was found in only one sample from the SEMP and thus no statistical support for host distribution could be obtained. The distributional patterns showed no overlap between “*Ca*. ^U^Cenporiarchaeum stylissum,” *Ca*. ^U^Nitrosopumilus hexadellus,” “*Ca*. ^U^Nitrosopumilus detritiferus,” and “*Ca*. ^U^N. cymbastelus” (Fig. 4; Fig. S2), and in most cases, each pair of thaumarchaeal species does not coexist within the same sponge species or sponge sample. The only exceptions are three sponge species (Cliona celata, Geodia barretti, and Petrosia ficiformis), where matches to both “*Ca*. ^U^N. hexadellus” and “*Ca*. ^U^N. detritiferus” were found.

10.1128/mSystems.00288-19.2 FIG S2 Venn diagram of the distribution of sponge-associated *Thaumarchaeota*. Download FIG S2, TIF file, 1.1 MB. Copyright © 2019 Zhang et al. 2019 Zhang et al. This content is distributed under the terms of the Creative Commons Attribution 4.0 International license.

Together, these data show that “*Ca*. ^U^Cenporiarchaeum stylissum” has a somewhat restricted host range by being predominantly found in *Stylissa* species and thus might have an obligate association with this sponge taxon. “*Ca*. ^U^Nitrosopumilus hexadellus” and “*Ca*. ^U^Nitrosopumilus detritiferus” occurred in a broader range of sponges, while “*Ca*. ^U^Nitrosopumilus cymbastelus” was frequently found outside sponges, indicating that these *Thaumarchaeota* have a more facultative relationship with particular sponge species or sponges in general.

A recent study demonstrated that sponge-associated symbiont communities are characterized by a combination of generalists and specialists (6). Generalists were defined as cosmopolitans that were not only present in a large number of sponge species but were also consistently present in a large fraction (>40%) of individuals of each host species. “*Ca*. ^U^Nitrosopumilus hexadellus” and “*Ca*. ^U^Nitrosopumilus detritiferus” clearly match this definition. “*Ca*. ^U^Cenporiarchaeum stylissum,” in contrast, appears to be more of a specialist, being restricted to a few closely related sponge taxa.

### Functional analysis showed features indicating adaptation to the sponge environment.

To gain further insights into thaumarchaeal adaptation to a sponge-associated lifestyle, indicator analysis (50) was undertaken for the relative abundance of orthologous groups (OGs) of proteins. Comparison of OGs has been extensively used to investigate the evolution of organisms and their potential functional adaptation to the environment or particular lifestyles (9, 32). OGs from sponge-associated “*Ca*. ^U^Cenporiarchaeum stylissum,” “*Ca*. ^U^Nitrosopumilus hexadellus,” “*Ca*. ^U^Nitrosopumilus detritiferus,” and closely related free-living counterparts were compared. “*Ca*. ^U^Nitrosopumilus cymbastelus” and “*Ca*. Cenarchaeum symbiosum” were excluded from the indicator analysis, as they are represented only by single genomes, thus precluding statistical analyses.

The total 43,542 predicted protein sequences of the thaumarchaeal species investigated here clustered with 40% identity and more than 80% coverage into 14,780 OGs. MAGs of the sponge-associated “*Ca*. ^U^Cenporiarchaeum stylissum” were compared to those from 15 free-living *Thaumarchaeota* (Table S1), and their combined 28,413 predicted protein sequences were contained in 4,748 OGs with at least two sequences per OG (Table 2). A total of 328 OGs in the indicator analysis had *P* values of <0.005 and were therefore indicative of either a sponge-associated or free-living lifestyle. Of these OGs, 248 were indicator OGs for sponge-associated genomes (Fig. S3), of which 100 could be assigned to archaeal Clusters of Orthologous Groups of proteins (arCOGs) (51, 52). The “*Ca*. ^U^Nitrosopumilus hexadellus” and the “*Ca*. ^U^Nitrosopumilus detritiferus” data sets with 163 and 140 indicator OGs, respectively, had 129 and 104 indicator OGs for a sponge-associated lifestyle, of which 51 OGs for each species could be assigned to arCOG functions, respectively (Table 2; Fig. S3). The genomes of “*Ca*. ^U^C. stylissum,” “*Ca*. ^U^N. hexadellus,” and “*Ca*. ^U^N. detritiferus” had 79, 34, and 36 indicator OGs with functions, respectively, which were significantly more abundant in all free-living thaumarchaeal genomes (Table 2; Fig. S3 and Table S3). These OGs with functions might be absent because they are no longer required for a sponge-associated lifestyle or simply due to the incompleteness of the MAGs.

**TABLE 2:** Indicator analysis of OGs and functional properties of “*Ca*. ^U^Cenporiarchaeum stylissum,” “*Ca*. ^U^Nitrosopumilus hexadellus,” and “*Ca*. ^U^Nitrosopumilus detritiferus”^*a*^

Characteristic	Parameter value for proposed species^*b*^		
	“*Ca*. ^U^C. stylissum”	“*Ca*. ^U^N. hexadellus”	“*Ca*. ^U^N. dedritiferus”
General characteristics			
Total no. of predicted protein sequences present in both SA and FL genomes	28,413	28,851	28,822
No. of OGs present in genomes	4,748	4,631	4,601

Indicator analysis			
No. of indicator OGs	328	163	140
No. of indicator OGs for SA genomes	248	129	104
No. of indicator OGs assigned to arCOG functions for SA genomes	100	51	51
No. of indicator OGs for FL genomes	80	34	36
No. of indicator OGs assigned to arCOG functions for FL genomes	79	34	36

a Abbreviations: SA, sponge-associated; FL, free-living. *P* < 0.005 for two sponge-associated *Thaumarchaeota* and 15 free-living *Thaumarchaeota*.

b The three proposed taxa are “*Candidatus* ^U^Nitrosopumilus hexadellus,” “*Candidatus* ^U^Nitrosopumilus detritiferus,” and “*Candidatus* ^U^Cenporiarchaeum stylissum.”

10.1128/mSystems.00288-19.3 FIG S3 Indicator analysis of OGs for “*Candidatus* ^U^Cenporiarchaeum stylissum” (A), “*Candidatus* ^U^Nitrosopumilus hexadellus” (B), and “*Candidatus* ^U^Nitrosopumilus detritiferus” (C) analyzed in comparison to other free-living counterparts based on relative abundance data. A total of 328, 163, and 140 OGs are present in the heat map showing significant differences (*P* value <0.005) between different lifestyles for “*Ca*. ^U^Cenporiarchaeum stylissum,” “*Ca*. ^U^Nitrosopumilus hexadellus,” and “*Ca*. ^U^Nitrosopumilus detritiferus,” respectively. The hierarchial trees of OGs and genomes are calculated based on Euclidian dissimilarities. Download FIG S3, TIF file, 2.7 MB. Copyright © 2019 Zhang et al. 2019 Zhang et al. This content is distributed under the terms of the Creative Commons Attribution 4.0 International license.

10.1128/mSystems.00288-19.8 TABLE S3 Comparison of the functional proteins and gene counts between sponge-associated “*Ca*. ^U^Cenporiarchaeum stylissum,” “*Ca*. ^U^Nitrosopumilus hexadellus,” and “*Ca*. ^U^Nitrosopumilus detritiferus.” Functional proteins were listed according to different groups that were classified by their presence in different *Thaumarchaeota* species. The values on the right side are the gene number of functional proteins present in corresponding genomes. Download Table S3, DOCX file, 0.1 MB. Copyright © 2019 Zhang et al. 2019 Zhang et al. This content is distributed under the terms of the Creative Commons Attribution 4.0 International license.

**FIG 5:**
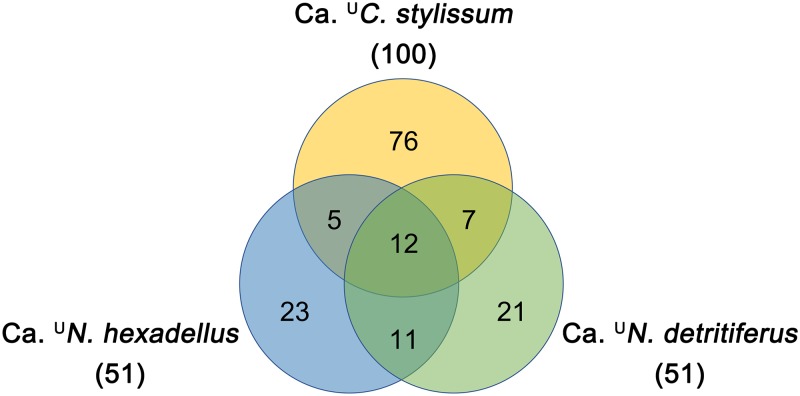
Venn diagram showing sponge-associated functions found in three thaumarchaeal species analyzed in comparison to other free-living *Thaumarchaeota*.

For all three species, OGs indicative of a sponge-associated lifestyle made up the majority (77.5% ± 4.7%) of all differential OGs (Fig. S3), providing support for the acquisition or enrichment of function in response to their particular symbiotic lifestyle. Twelve of these sponge-associated OGs, which could be assigned to arCOG functions, were found in all three species. “*Ca*. ^U^Cenporiarchaeum stylissum” had more sponge-associated functions than “*Ca*. ^U^Nitrosopumilus hexadellus” or “*Ca*. ^U^Nitrosopumilus detritiferus” did (Fig. 5), indicating different degrees of functional or evolutionary adaptation to the sponge environment.

### Shared OGs with functions involve metabolic and defense processes.

**FIG 6:**
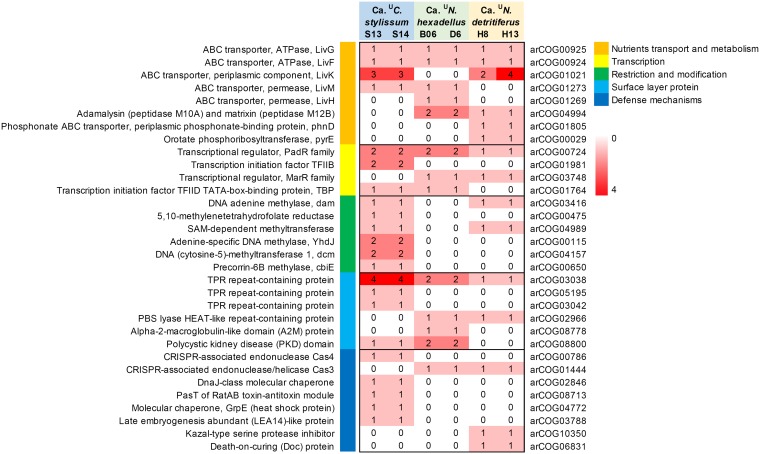
Comparison of sponge-associated functions in “*Ca*. ^U^Cenporiarchaeum stylissum,” “*Ca*. ^U^Nitrosopumilus hexadellus,” and “*Ca*. ^U^Nitrosopumilus detritiferus” using arCOG-based annotation. Details of additional functions are given in Table S3 in the supplemental material.

The 12 functions that were encoded by the indicator OG genes shared by “*Ca*. ^U^Cenporiarchaeum stylissum,” “*Ca*. ^U^Nitrosopumilus hexadellus,” and “*Ca*. ^U^Nitrosopumilus detritiferus” include Liv-type ATP-binding cassette (ABC) transporter ATPases (arCOG00924 and arCOG00925), a phenolic acid decarboxylase regulator (PadR)-like transcriptional regulator (arCOG00724), a tetratricopeptide repeat (TPR)-containing protein (arCOG03038) and enzymes Cas3 and Cas4 (arCOG01444 or arCOG00786) (Fig. 6; Table S3).

The cytoplasmic ATPases LivG (arCOG00925) and LivF (arCOG00924) belong to ABC-type transporters for branched-chain amino acids (Leucine-isoleucine-valine [Liv]) (53). These transporters also contain membrane-integrated permeases, for which different types (LivK, -M, and -H) were present in three sponge-associated *Thaumarchaeota*. In Escherichia coli, different types of permeases have been shown to contribute to the specificity of the transport system (54). In addition, “*Ca*. ^U^Cenporiarchaeum stylissum” and “*Ca*. ^U^Nitrosopumilus detritiferus” had high copy numbers for a unique OG that was annotated as a LivK-type periplasmic component (arCOG01201), which is involved in substrate binding during import (55). This indicates that import of branched-chain amino acids might be an import feature for sponge-associated *Thaumarchaeota*, which is consistent with other studies showing that ABC transporters might have a role in scavenging nutrients for bacteria within the sponge environment (12, 32, 33, 56, 57).

The PadR family (arCOG00724) comprises a diverse array of transcriptional regulators involved, for example, in detoxification of phenolic acids (58, 59), expression of multidrug efflux pumps (60), or virulence gene expression (61). Its exact role in sponge-associated *Thaumarchaeota* is therefore not clear. TPR-containing protein can mediate protein-protein interactions in eukaryotes (62) and might be used by sponge-associated bacteria to interfere with phagosome processing (63).

Clustered regularly interspaced short palindromic repeats (CRISPR) and their associated Cas proteins constitute an adaptive immune system found in many prokaryotic genomes that provides protection against mobile genetic elements (MGEs), including viruses, transposable elements, and conjugative plasmids (64, 65). Cas3 has been proposed to play a key role in the CRISPR mechanism through direct cleavage of invasive nucleic acids (66, 67). Cas4 belongs to the RecB family of exonuclease, which is suggestive of DNA binding activity. The *cas4* gene has been reported to be strictly associated with CRISPR elements (65) and appears to be less conserved than other *cas* genes, such as *cas1* and *cas2* (68, 69). In further support of the existence of CRISPR mechanisms in the sponge-associated *Thaumarchaeota*, we found CRISPR arrays and *cas* genes on the same genomic scaffolds for “*Ca*. ^U^Cenporiarchaeum stylissum” MAG ST14 and “*Ca*. ^U^Nitrosopumilus hexadellus” MAG B06 (Fig. S4). CRISPRs and *cas* genes could also be found in the genomes of “*Ca*. ^U^Nitrosopumilus detritiferus” but were located on different scaffolds, most likely due to incomplete assembly. Together, these results indicate the existence of some common features, such as amino acid transport as well as DNA defense, in the adaptation of thaumarchaeal species to the sponge environment. These findings are similar to recent findings that CRISPR and other defense-related features were found enriched in sponge-associated bacterial symbionts (70).

10.1128/mSystems.00288-19.4 FIG S4 Schematic diagram of the CRISPR arrays and *cas* genes in “*Ca*. ^U^Cenporiarchaeum stylissum” (A) and “*Ca*. ^U^Nitrosopumilus hexadellus” (B). They were found on scaffold 877 of “*Ca*. ^U^Cenporiarchaeum stylissum” MAG ST14 and scaffold 7 of “*Ca*. ^U^Nitrosopumilus hexadellus” MAG B06, respectively. *cas* genes are indicated with arrows in different colors, and the CRISPR array is shown with blue striped boxes. The numbers under genes indicate their DNA locus. Download FIG S4, TIF file, 0.8 MB. Copyright © 2019 Zhang et al. 2019 Zhang et al. This content is distributed under the terms of the Creative Commons Attribution 4.0 International license.

### Shared and unique OGs of the generalist symbionts “*Ca*. ^U^Nitrosopumilus hexadellus” and “*Ca*. ^U^Nitrosopumilus detritiferus.”

Almost half (44.7% ± 0.1%) of the functions encoded by sponge-associated OGs in “*Ca*. ^U^Nitrosopumilus hexadellus” are the same as in “*Ca*. ^U^Nitrosopumilus detritiferus,” including a unique PBS lyase HEAT-like repeat domain (arCOG02966) (71–73). Proteins with this repeat domain have been reported to inactivate exogenous proteases (73) and therefore could mediate evasion of a host’s innate defense systems and resistance against phagocytosis. Both “*Ca*. ^U^N. hexadellus” and “*Ca*. ^U^N. detritiferus” also carried unique genes encoding components annotated as adamalysin (peptidase M10A) and matrixin (peptidase M12B) (arCOG04994), indicating that the two archaea have similar opportunities for the degradation of proteins and uptake of amino acids, which might be related to the existence of an extracellular protein matrix of their hosts.

“*Ca*. ^U^Nitrosopumilus hexadellus” and *Ca*. ^U^Nitrosopumilus detritiferus” also have unique, annotated OGs that discriminate their genomes. For example, “*Ca*. ^U^N. hexadellus” contains a unique OG annotated as polycystic kidney disease (PKD) domain (arCOG08800) and another unique OG annotated as an ELP that consists of repeats of the alpha-2-macroglobulin-like domain (A2M) (arCOG08778). PKD domains have been previously detected in archaeal surface layer proteins (74), and both PKD and A2M have been suggested to have a role in interacting with the cell surface proteins of metazoans (73). An OG that was annotated as transcription initiation factor TFIID TATA box-binding protein (TBP) (arCOG01764) was also unique to “*Ca*. ^U^N. hexadellus,” potentially playing a role in sensing and responding to the specific environmental conditions given in its sponge host.

As for features that distinguish “*Ca*. ^U^Nitrosopumilus detritiferus” from “*Ca*. ^U^Nitrosopumilus hexadellus,” we found OGs that encode DNA adenine methylase (Dam) (arCOG03416) and *S*-adenosylmethionine (SAM)-dependent methyltransferase (arCOG04989). Dams and SAM-dependent methyltransferases and associated cognate endonucleases form restriction-modification (R-M) systems that control the invasion of foreign DNA (75). Dams were enriched in both “*Ca*. ^U^Cenporiarchaeum stylissum” and “*Ca*. ^U^N. hexadellus” and were found next to the cognate endonuclease Endonuc-EcoRV (Pfam accession number PF09233) in “*Ca*. ^U^C. stylissum” MAG ST14, strongly suggesting they are part of a functional R-M systems (76, 77). Protection against foreign DNA has previously been hypothesized to be an important feature of sponge-associated microbial communities that must maintain genomic integrity in an environment with a constant influx of biological material, including DNA and viruses, derived from the sponge’s filter-feeding activity (32, 70). “*Ca*. ^U^N. detritiferus” also carries genes that encode unique OGs that were annotated as prophage death-on-curing (Doc) protein (arCOG06831) and the kazal-type serine protease inhibitor (arCOG10350), which have been reported to play important roles in bacterial stress response (78) and defense against proteinases from pathogenic bacteria (79). Together with the CRISPR mentioned above, these results imply a general need for defense mechanisms in “*Ca*. ^U^N. detritiferus.” In addition, one OG annotated as a periplasmic binding protein of a phosphonate ABC transporter (PhnD) (arCOG01805) was exclusively found in “*Ca*. ^U^N. detritiferus.” The ATPase (PhnC) and permease (PhnE) were also found in “*Ca*. ^U^N. detritiferus” MAG H8. The *phn* genes are generally induced under phosphate limitation (80), perhaps indicating a potential adaptation of “*Ca*. ^U^N. detritiferus” to a specific nutritional environment (i.e., limited phosphate) in *Hexadella* cf. *detritifera*.

### Unique OGs with functional features in the specialist “*Ca*. ^U^Cenporiarchaeum stylissum.”

“*Ca*. ^U^Cenporiarchaeum stylissum” contains unique OGs that were annotated as methylases (YhdJ, arCOG00115; CbiE, arCOG00650) and methyltransferase (Dcm, arCOG04157) belonging to R-M systems, the heat shock protein GrpE (arCOG04772), and a DnaJ-class molecular chaperone (arCOG02846), a late embryogenesis abundant (LEA14)-like protein (arCOG03788), and proteins involved in a toxin-antitoxin (TA) module, including a persistence and stress resistance toxin PasT (arCOG08713). Heat shock proteins and chaperones protect other proteins from irreversible aggregation during synthesis and in times of cellular stress (81–83), and this has been postulated as an evolutionary adaptation in the symbiotic lifestyle of dinoflagellates (84). “*Ca*. ^U^Cenporiarchaeum stylissum” has three OGs assigned to arCOG02846 (DnaJ-class molecular chaperone), of which one is unique to the organism. This unique copy could represent a specific functional adaptation or experience specific gene expression under conditions that are important for “*Ca*. ^U^Cenporiarchaeum stylissum.” “*Ca*. ^U^Cenporiarchaeum stylissum” also has only one OG that was annotated as GrpE (arCOG04772), which is divergent from the OG with a gene that encodes GrpE in the other sponge-associated or free-living *Thaumarchaea*. It is unclear why this OG has diverged so much from those found in the other closely related archaea, but this could reflect a potential functional adaptation. The LEA14-like protein is thought to be associated with archaeal stress response and functions either in archaeal defense or by interacting with host signaling pathways (85). TA systems are prevalent in many bacterial genomes and contribute to biofilm and persister cell formation (86, 87). Specifically, in pathogenic Escherichia coli, the PasT of TA systems increased its antibiotic stress resistance (88). Such a defense mechanism might also be useful for sponge-associated *Thaumarchaeota* given a large number of chemical antagonists produced by sponges (89). Interestingly, “*Ca*. ^U^Cenporiarchaeum stylissum” also had a unique set of significantly enriched OGs that were annotated as TPRs (arCOG05195 and arCOG03042), which suggests a different kind of molecular interaction with the sponge host than what occurs in “*Ca*. ^U^Nitrosopumilus hexadellus” and “*Ca*. ^U^Nitrosopumilus detritiferus” (63).

### Summary.

In our study, three new sponge-associated thaumarchaeal species were described, and we propose them to be specialist (“*Ca*. ^U^Cenporiarchaeum stylissum”) or generalist (“*Ca*. ^U^Nitrosopumilus hexadellus” and “*Ca*. ^U^Nitrosopumilus detritiferus”) species based on their observed host distribution. The unique and shared genetic characteristics of “*Ca*. ^U^Cenporiarchaeum stylissum,” “*Ca*. ^U^Nitrosopumilus hexadellus,” and “*Ca*. ^U^Nitrosopumilus detritiferus” have highlighted several genomic strategies for a sponge-associated lifestyle. Genomic traits found in all three species (e.g., CRISPR, TPRs) indicate a functional convergence reflecting general adaptation to the sponge environment. The unique characteristics for the specific thaumarchaeal taxa studied here highlight how generalists and specialists could be at different stages of evolutionary adaptation, employ different ecological strategies, and/or are exposed to different environmental conditions within the sponge hosts. Our study therefore provides new evolutionary and ecological insights into the symbiosis between *Thaumarchaeota* and marine sponges.

## MATERIALS AND METHODS

### Sample collection and sequencing.

Four individual deep-sea sponge specimens were collected from three stations in the North Atlantic Ocean (see Fig. S5 in the supplemental material; map drawn with ODV software [90]). Sponge sample B0601MIN (B06) and H8 were collected from locations close to Mingulay, Scotland, and the Celtic Sea, France, respectively, while sponge sample D6ROC (D6) and H13 were sampled from the Irish Sea, Ireland. Phylogenetic analysis showed that these four sponges belong to the genus *Hexadella* but likely represent two different species within the genus with sample B06 and D6 being *Hexadella detritifera* and sample H8 and H13 belonging to *Hexadella* cf. *detritifera* (91, 122). Total genomic DNA was extracted from sponge samples using the MoBio PowerPlant DNA isolation kit following the manufacturer’s instructions (MO BIO Laboratories, CA, USA). We used a Covaris S series sonicator to shear DNA to ∼175-bp fragments and constructed metagenomic libraries using the Ovation Ultralow Library DR multiplex system (Nugen Redwood City, CA, USA) following the manufacturer’s instructions. Metagenomic sequencing was conducted on the Illumina HiSeq 1000 platform with up to 2 × 113 bp chemistry at the W.M. Keck sequencing facility at the Marine Biological Laboratory (Woods Hole, MA, USA).

10.1128/mSystems.00288-19.5 FIG S5 Station map in the North Atlantic Ocean. The map was drawn with the ODV software (90). Download FIG S5, TIF file, 1.3 MB. Copyright © 2019 Zhang et al. 2019 Zhang et al. This content is distributed under the terms of the Creative Commons Attribution 4.0 International license.

Three samples (S13, S14, and S15) of the sponge *Stylissa flabelliformis* were collected from the Davies Reef, Great Barrier Reef, Australia. Tissue samples were frozen in liquid nitrogen and stored at –80°C. Each sample was homogenized in collagenase and then centrifuged for microbial cell collection. Microbial community DNA was extracted with a Qiagen UltraClean Microbial DNA Isolation kit (92). Extracted DNA was further purified using the ZymoResearch (CA, USA) purification kit (Genome DNA clean and concentrator). Nextera XT library preparation was performed on all samples at the Ramaciotti Centre for Genomics (University of New South Wales, Australia), and samples were then sequenced on the Illumina MiSeq platform with 2 × 250-bp chemistry.

Further details about sample collection are presented in Table S4 and Fig. S5 in the supplemental material.

10.1128/mSystems.00288-19.9 TABLE S4 Details of sample collection. Download Table S4, DOCX file, 0.01 MB. Copyright © 2019 Zhang et al. 2019 Zhang et al. This content is distributed under the terms of the Creative Commons Attribution 4.0 International license.

### Metagenome-assembled genome reconstruction.

Metagenomic sequences obtained from individual sponges were analyzed separately to reconstruct archaeal metagenome-assembled genomes (MAGs) for comparative analysis. Paired-end reads were quality filtered and trimmed with Trimmomatic v.0.33 using the following parameters: “SLIDINGWINGDOW:6:30 MINGLEN:50” (93). Reads were assembled using IDBA_UD v.1.1.1 with the kmer size from 20 to 100 bp and an interval of 20 (94). Only contigs larger than 2.0 kbp were kept, and contig coverage was calculated by mapping reads back to the contigs using the end-to-end option of Bowtie2 v.2.2.9 (95). Metagenome binning was performed using both MetaBAT v.0.32.4 (96) and MyCC (97), and bins were then refined using Binning_refiner (98). MAG quality was assessed by CheckM based on the presence of 146 single-copy marker genes, which were grouped into 104 lineage-specific marker sets from 207 archaeal genomes (41). The genome size was estimated by dividing the bin size by its estimated completeness. High heterogeneity was evident in some of the bins due to the high coverage (e.g., the sequencing depth of data set D6 was about 361×). In these cases, bins with high quality were obtained by subsampling the metagenomic reads to reduce the coverage followed by assembly and binning as described above.

### Phylogenetic analysis.

The 16S rRNA gene sequences were reconstructed from metagenomic reads using MATAM (40), and their taxonomical information was obtained by alignment to the SILVA database v1.2.11 (99, 100). Reconstructed thaumarchaeal 16S rRNA gene sequences were added to the MAG, if they had a sequence similarity of ≥98.6% (101) and an alignment length of more than 400 bp with any scaffold within the MAG. All aligned thaumarchaeal 16S rRNA genes were then subjected to a BLASTN search (102, 103) against the nonredundant nucleotide (NT) database at the NCBI on 2 December 2017, and top hits with closed genomes were aligned in order to determine sequence similarities (104, 105). In addition, another sponge-associated thaumarchaeal genome bin (Ga0078905) with corresponding 16S rRNA genes assembled from metagenomic data sets from the sponge *Cymbastela concentrica* was included (33). 16S rRNA gene sequences of >1,000 bp were aligned using MAFFT v7.310 (106), and a maximum-likelihood tree was calculated using RAxML v.8.2.10 with a GTRGAMMA model and 1,000 bootstraps (107). The tree was visualized using iTOL (108) and rooted with the sequence of two Aigarchaeota (“*Candidatus* Caldiarchaeum subterraneum” NC_022786.1 and an unclassified Aigarchaeota Ga0180309_101) and one unclassified *Thaumarchaeota* (Ga0181444_1001) as an outgroup (109, 110). The 16S rRNA genes of the new MAGs were also searched against the Sponge Earth Microbiome Project (SEMP) database (http://www.spongeemp.com) (49), and the search results were manually curated to remove hits against biofilm samples, whose exact nature were unclear.

For further taxonomic classification, pairwise average amino acid identity (AAI) distance between new MAGs and 74 thaumarchaeal reference genomes was calculated with the Microbial Genomes Atlas (111). Pairwise AAI distance between the new MAGs and the 16 most similar closed reference genomes was also calculated using CompareM (https://github.com/dparks1134/CompareM). The SCG tree for all input genomes was inferred from the concatenation of 122 archaeal single-copy proteins identified as being present in ≥90% of archaeal genomes and, when present, being present in a single copy in ≥95% of the genomes (112). Predicted protein sequences for the input genomes were searched against the PFAM v31.0 (113) and TIGRFAM v14.0 (114) hmm profiles of these SCG proteins using HMMER v3.1b2 (115). Protein sequences for each hmm profile were then individually aligned with MAFFT v7.310 and concatenated into a multiple-sequence alignment (MSA). A phylogenetic tree was then generated by RAxML v.8.2.10 with a PROTGAMMAWAG model and 1,000 bootstraps and visualized as well as rooted as described above.

### Gene annotation and comparison.

Prodigal, as implemented in Prokka, was used to predict open reading frames (ORF) in the MAGs using the “metagenome” setting and specifying the kingdom as “Archaea” (116, 117). All predicted protein sequences were clustered into orthologous groups (OGs) using the OrthoMCL v1.4 clustering algorithm (118) as implemented in the program get_homologues v18092017 (119). Bidirectional BLAST searches were filtered with an E value of 10^−05^ as well as >40% alignment identity over 80% alignment coverage, which has been demonstrated to have a probability of >90% that the sequences in OGs are also homologous (120). The longest sequence in each OG is then used for functional annotation. OGs that are characteristic of sponge-associated and free-living lifestyle were identified using indicator analysis on relative abundance data (50). Abundance data for OGs were also visualized using the R package “pheatmap” (121). OGs that displayed significant differences between lifestyles were further annotated by searching them with BLASTP and an E value of 10^−4^ against the archaeal Clusters of Orthologous Groups of proteins (arCOGs) database released in December 2014 (51, 52). Some functional names of clusters were corrected by annotation results against the Clusters of Orthologous Groups of proteins (COGs) and KEGG Orthology (KO) databases.

### Data availability.

Sequences from this project have been deposited at the GenBank database under the genome accession numbers RHFA00000000, RHEZ00000000, and RHEY00000000 for “*Ca*. ^U^Cenporiarchaeum stylissum,” RHFD00000000 and RHFE00000000 for “*Ca*. ^U^Nitrosopumilus hexadellus,” and RHFB00000000 and RHFC00000000 for “*Ca*. ^U^Nitrosopumilus detritiferus.”
