# Supplementary figures and images for: Comparative Genomics Reveals Ecological and Evolutionary Insights into Sponge-Associated Thaumarchaeota
Source: mSystems. 2019 Aug 13;4(4):e00288-19. doi: 10.1128/mSystems.00288-19 (PMC6697440; doi:10.1128/mSystems.00288-19)

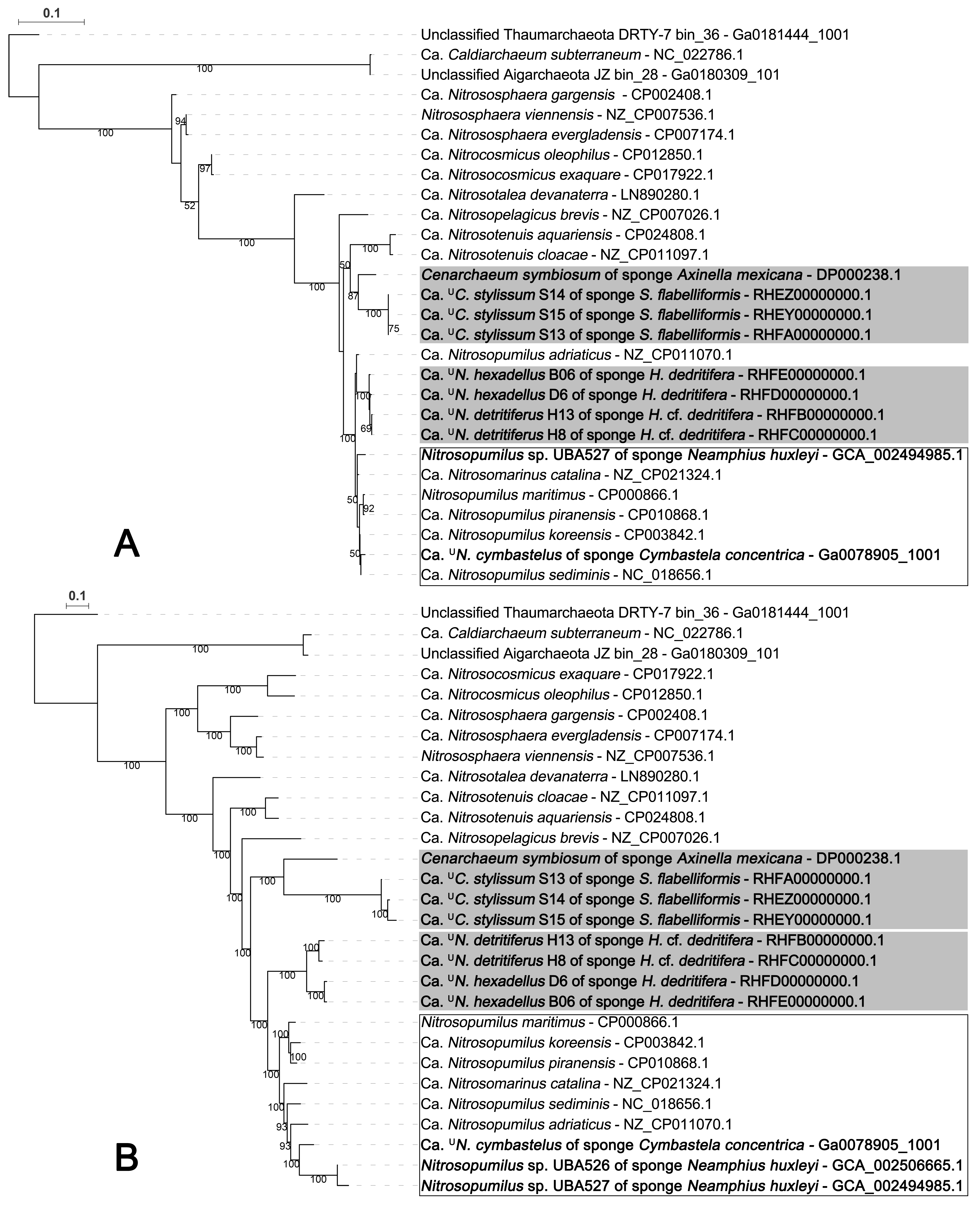

Supplement: FIG S1 [file mSystems.00288-19-sf001.tif]

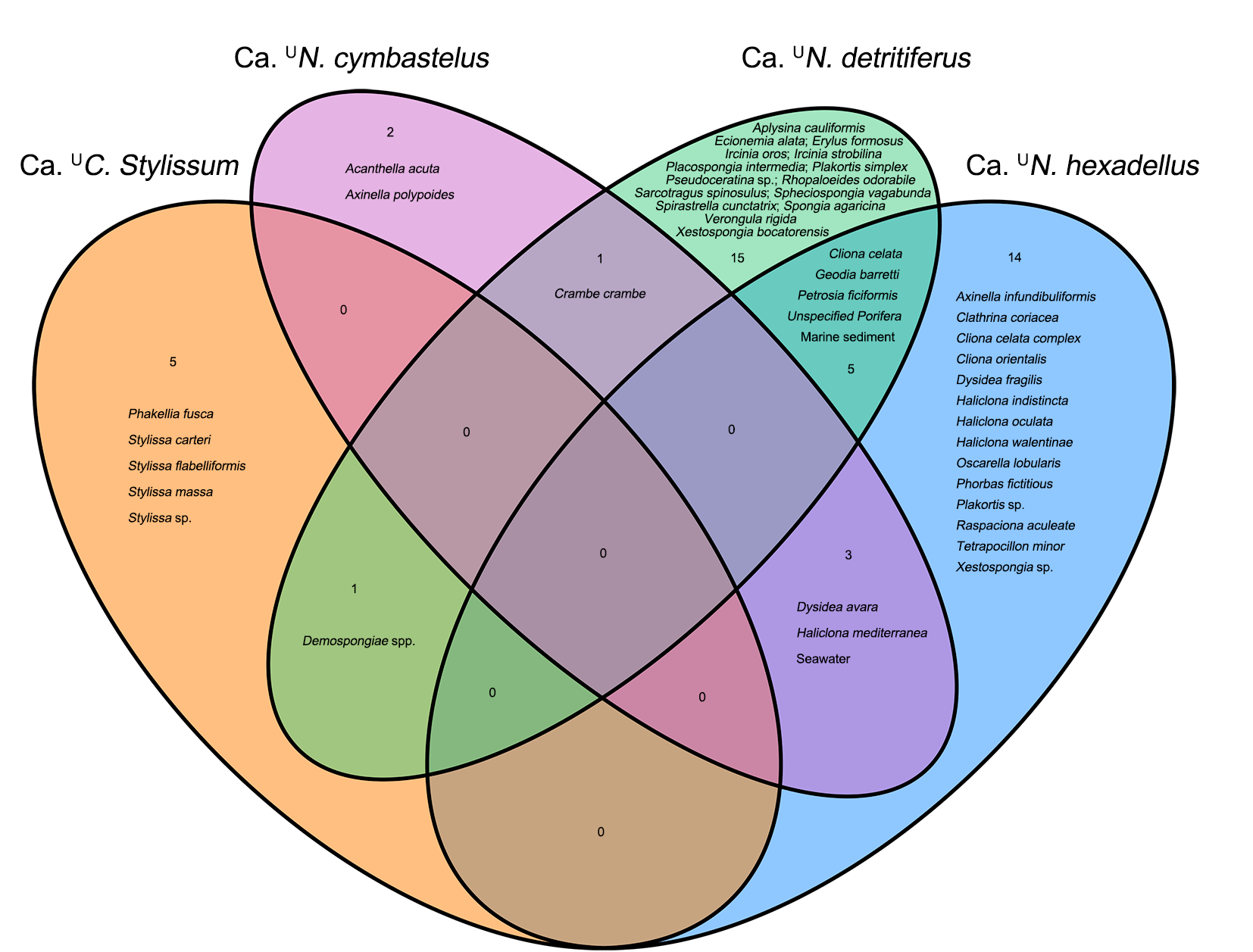

Supplement: FIG S2 [file mSystems.00288-19-sf002.tif]

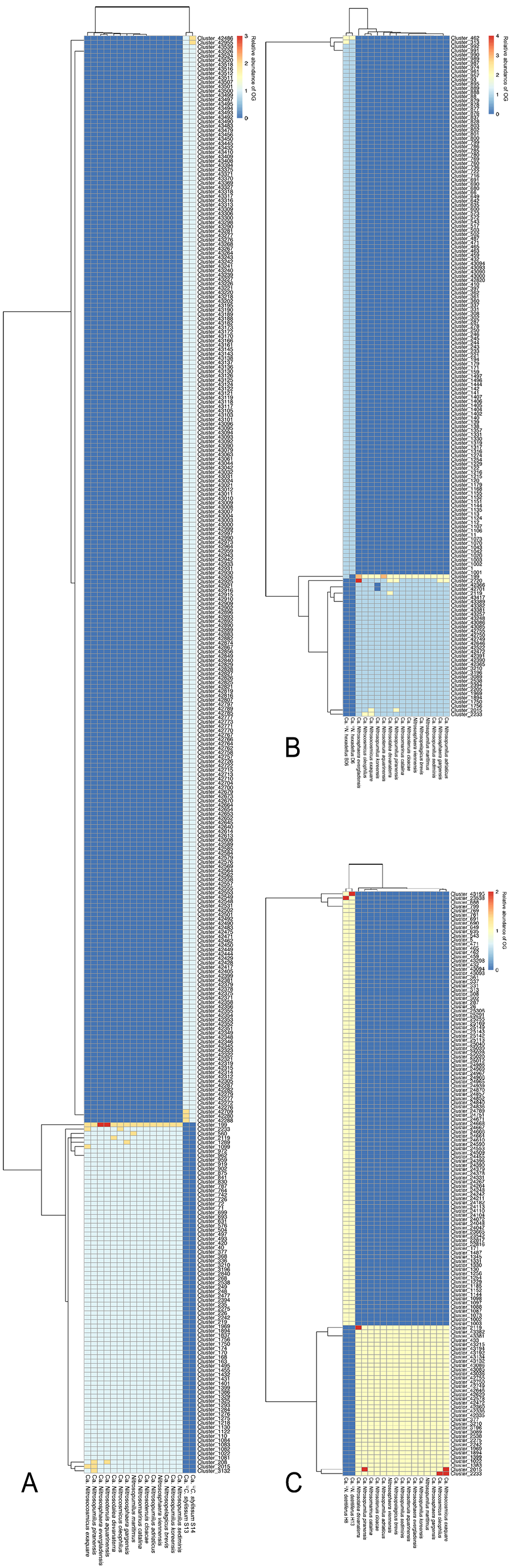

Supplement: FIG S3 [file mSystems.00288-19-sf003.tif]

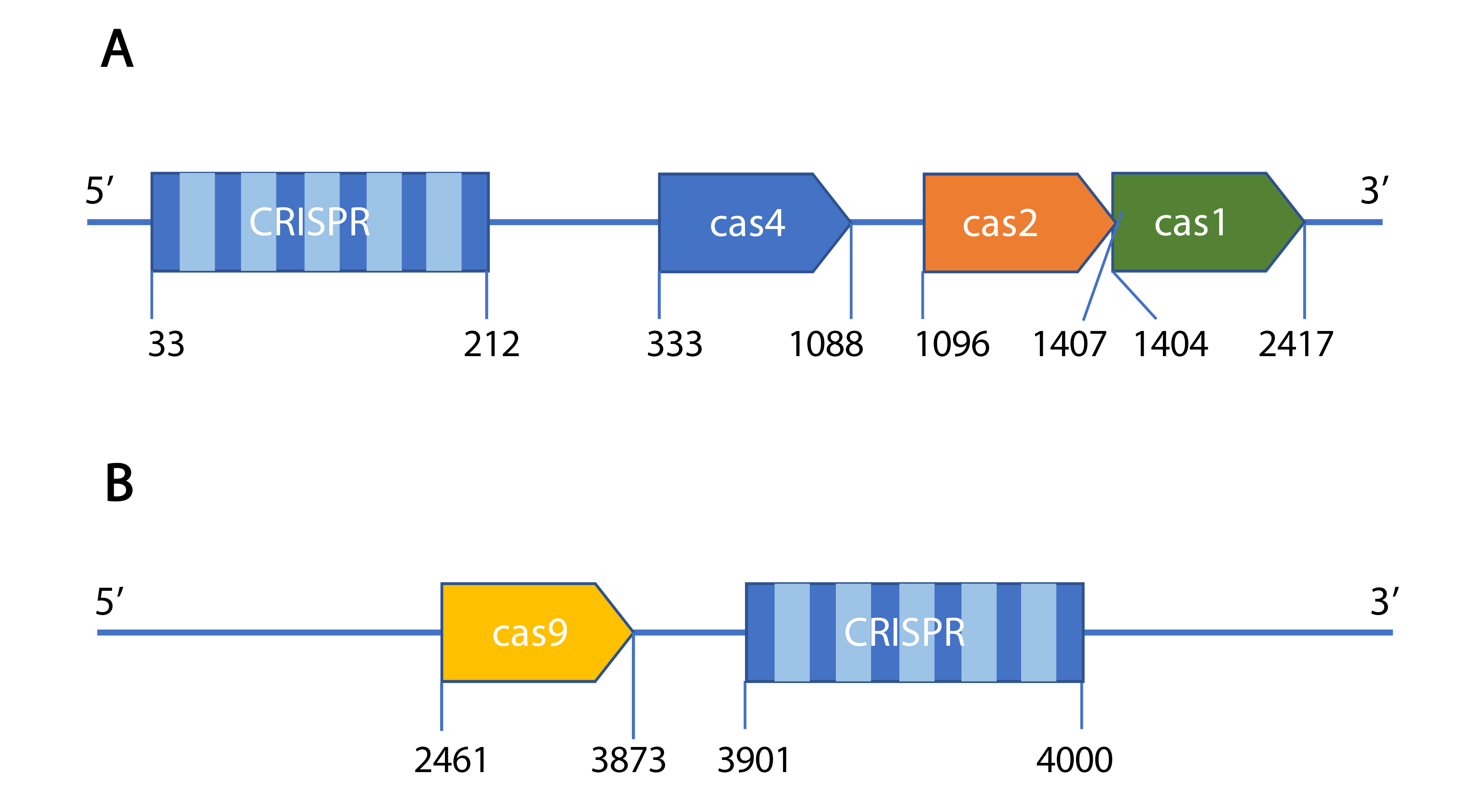

Supplement: FIG S4 [file mSystems.00288-19-sf004.tif]

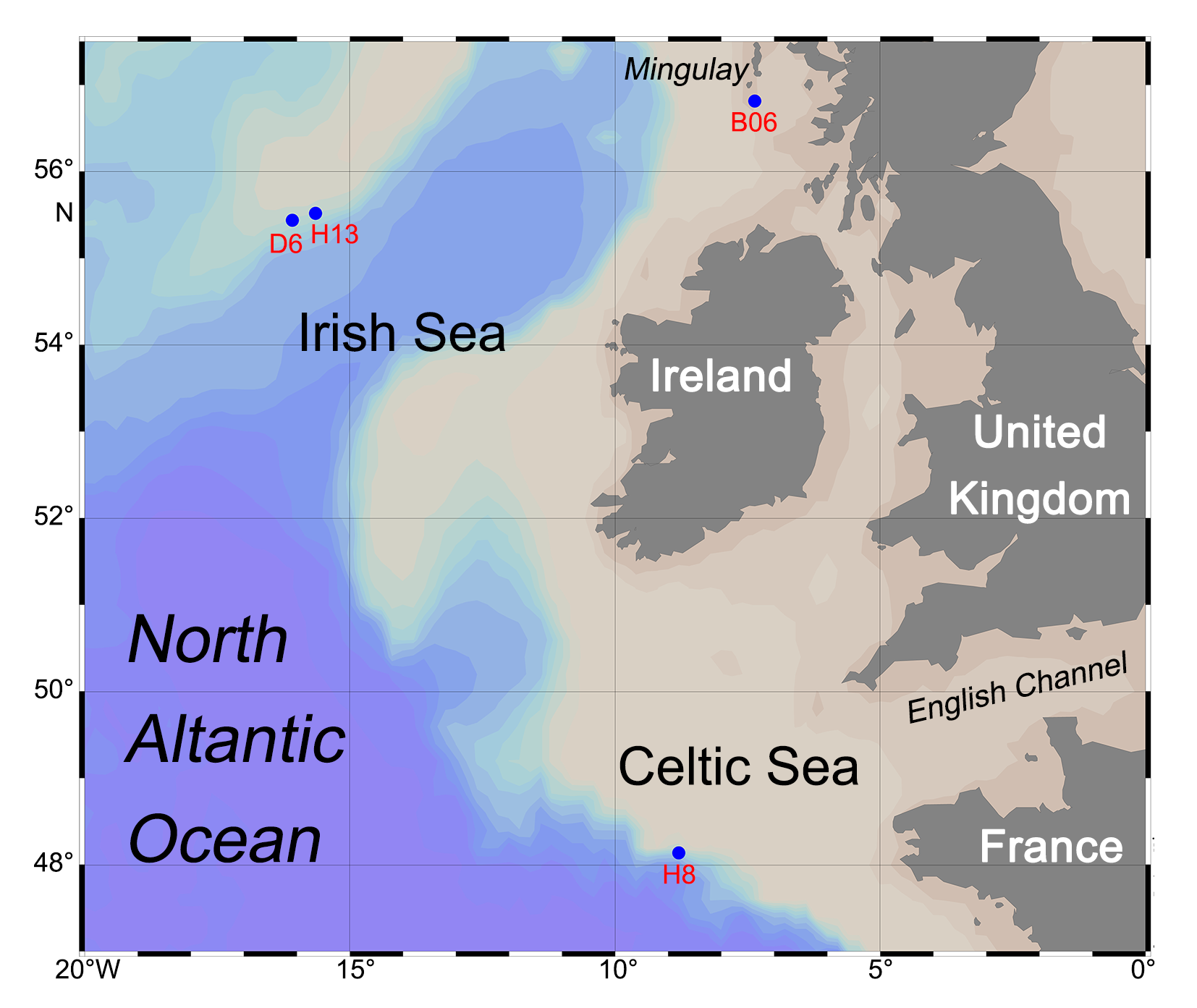

Supplement: FIG S5 [file mSystems.00288-19-sf005.tif]
