# Supplementary material for: Comparative Genomics Reveals Ecological and Evolutionary Insights into Sponge-Associated Thaumarchaeota
Source: mSystems. 2019 Aug 13;4(4):e00288-19. doi: 10.1128/mSystems.00288-19 (PMC6697440; doi:10.1128/mSystems.00288-19)
Supplement: TABLE S1 [file mSystems.00288-19-st001.docx]

| **Genome name** | **Strain** | **GenBank ID** | **NCBI accession number** | **Habitat** | **Biotic relationships** | **Taxon ID** | **GC%** | **Genome size/Mbp** | **Relevant publication** |
| --- | --- | --- | --- | --- | --- | --- | --- | --- | --- |
| *Nitrosopumilus maritimus* | SCM1 | CP000866.1 | CP000866 | Seawater of Seattle Aquarium, Seattle, WA, USA | Free-living | 641228499 | 34.17 | 1.57 | (14) |
| Candidatus *Nitrososphaera gargensis* | Ga9.2 | CP002408.1 | CP002408 | Hot spring, Baikal Rift Zone, southeastern Russia, Russia | Free-living | 2510065023 | 48.35 | 2.7 | (18) |
| Candidatus *Nitrosopumilus koreensis* | AR1 | CP003842.1 | CP003842 | 78-m-deep marine sediment off Svalbard, the Arctic Circle | Free-living | 2518645532 | 34.18 | 1.56 | (27) |
| Candidatus *Nitrososphaera evergladensis* | SR1 | CP007174.1 | CP007174 | Agricultural soil, histosol from the Everglades agricultural area, Florida America | Free-living | 2585427666 | 50.14 | 2.82 | (24) |
| Candidatus *Nitrosopumilus piranensis* | D3C | CP010868.1 | CP010868 | Coastal surface water, Northern Adriatic Sea | Free-living | 2627853696 | 33.82 | 1.63 | (16) |
| Candidatus *Nitrocosmicus oleophilus* | MY3 | CP012850.1 | CP012850 | Terrestrial, surface sediment | Free-living | 2657244923 | 34.14 | 3.27 | (25) |
| Candidatus *Nitrosocosmicus exaquare* | G61 | CP017922.1 | CP017922 | Industrial wastewater, Guelph, Ontario, Canada | Free-living | 2718217702 | 33.94 | 2.85 | (21) |
| Candidatus *Nitrosotenuis aquariensis* | AQ6f | CP024808.1 | CP024808 | Freshwater aquarium biofilters | Free-living | 2651869505 | 42.18 | 1.62 | (19) |
| Candidatus *Nitrosotalea devanaterra* | NDEV1 | LN890280.1 | LN890280 | Acidic agricultural soil, Craibstone, Aberdeen, Scotland, United Kingdom | Free-living | 2757320681 | 37.07 | 1.72 | (22) |
| Candidatus *Nitrosopumilus sediminis* | AR2 | NC_018656.1 | NC_018656 | Marine sediment off Svalbard, the Arctic Circle | Free-living | 2518645576 | 33.59 | 1.61 | (26) |
| Candidatus *Nitrosopelagicus brevis* | CN25 | NZ_CP007026.1 | NZ_CP007026 | Surface seawater, open ocean | Free-living | 2630968793 | 33.16 | 1.18 | (15) |
| *Nitrososphaera viennensis* | EN76 | NZ_CP007536.1 | NZ_CP007536 | Garden soil, Vienna Austria | Free-living | 2585427612 | 52.72 | 2.41 | (23) |
| Candidatus *Nitrosopumilus adriaticus* | NF5 | NZ_CP011070.1 | NZ_CP011070 | Coastal surface water, Northern Adriatic Sea | Free-living | 2627854092 | 33.41 | 1.72 | (16) |
| Candidatus *Nitrosotenuis cloacae* | SAT1 | NZ_CP011097.1 | NZ_CP011097 | Industrial wastewater, Tianjin China | Free-living | 2630968650 | 41 | 1.55 | (20) |
| Candidatus *Nitrosomarinus catalina* | SPOT01 | NZ_CP021324.1 | NZ_CP021324 | Ocean water, temperate Pacific waters off California | Free-living | 2757320762 | 31.44 | 1.3 | (17) |
| *Cenarchaeum symbiosum* | A | DP000238.1 | DP000238 | Marine sponge tissue, *Axinella mexicana* | Sponge-associated | 641522613 | 57.37 | 1.95 | (30) |
