# Supplementary material for: Comparative Genomics Reveals Ecological and Evolutionary Insights into Sponge-Associated Thaumarchaeota
Source: mSystems. 2019 Aug 13;4(4):e00288-19. doi: 10.1128/mSystems.00288-19 (PMC6697440; doi:10.1128/mSystems.00288-19)
Supplement: TABLE S2 [file mSystems.00288-19-st002.docx]

| **Species and sample name** | **Host name** | **Samples positive/Samples in database** | **Binomial p-value** | **Rank-sum p-value** |
| --- | --- | --- | --- | --- |
| **Ca. ^U^*C. stylissum*** | *Demospongiae* spp. | 7/79 | 0.015489 | 0.005275 |
|  | *Phakellia fusca* | 4/4 | 0.000001 | < 0.000001 |
|  | *Stylissa carteri* | 5/5 | < 0.000001 | < 0.000001 |
|  | *Stylissa flabelliformis* | 29/30 | < 0.000001 | < 0.000001 |
|  | *Stylissa massa* | 38/38 | < 0.000001 | < 0.000001 |
|  | *Stylissa* sp. | 4/4 | 0.000001 | < 0.000001 |
| **Ca. ^U^*N. hexadellus*** | *Axinella infundibuliformis* | 11/12 | 0.000007 | < 0.000001 |
|  | *Clathrina coriacea* | 5/6 | 0.00761 | 0.000027 |
|  | *Cliona celata* | 11/14 | 0.000114 | 0.000023 |
|  | *Cliona celata complex* | 7/7 | 0.000127 | 0.000015 |
|  | *Cliona orientalis* | 20/31 | 0.000022 | 0.000149 |
|  | *Dysidea avara* | 32/58 | 0.00001 | 0.000026 |
|  | *Dysidea fragilis* | 14/20 | 0.000105 | 0.000205 |
|  | *Geodia barretti* | 80/117 | < 0.000001 | < 0.000001 |
|  | *Haliclona indistincta* | 5/6 | 0.00761 | 0.001197 |
|  | *Haliclona mediterranea* | 5/5 | 0.00165 | 0.000366 |
|  | *Haliclona oculata* | 11/25 | 0.060264 | 0.062362 |
|  | *Haliclona walentinae* | 5/9 | 0.073738 | 0.052527 |
|  | *Oscarella lobularis* | 6/13 | 0.122859 | 0.032899 |
|  | *Petrosia ficiformis* | 47/101 | 0.000045 | < 0.000001 |
|  | *Phorbas fictitious* | 12/23 | 0.011416 | 0.068714 |
|  | *Plakortis* sp. | 24/31 | < 0.000001 | < 0.000001 |
|  | *Raspaciona aculeate* | 4/6 | 0.054123 | 0.090951 |
|  | *Tetrapocillon minor* | 6/6 | 0.000458 | 0.000149 |
|  | *Xestospongia* sp. | 16/38 | 0.040254 | 0.049792 |
|  | Unspecified Porifera | 93/250 | 0.000745 | 0.001989 |
|  | Seawater | 191/310 | < 0.000001 | < 0.000001 |
|  | Marine sediment | 53/57 | < 0.000001 | < 0.000001 |
| **Ca. ^U^*N. cymbastelus*** | *Acanthella acuta* | 6/6 | < 0.000001 | < 0.000001 |
|  | *Axinella polypoides* | 8/8 | < 0.000001 | < 0.000001 |
|  | *Crambe crambe* | 6/52 | 0.001829 | 0.000027 |
|  | *Dysidea avara* | 9/58 | 0.000013 | < 0.000001 |
|  | *Haliclona mediterranea* | 4/5 | 0.000002 | < 0.000001 |
|  | Seawater | 31/310 | < 0.000001 | < 0.000001 |
| **Ca. ^U^*N.* *detritiferus*** | *Aplysina cauliformis* | 6/9 | 0.003677 | 0.000884 |
|  | *Cliona celata* | 6/14 | 0.050854 | 0.07433 |
|  | *Crambe crambe* | 19/52 | 0.006149 | 0.016182 |
|  | *Demospongiae* spp. | 32/79 | 0.000048 | 0.000019 |
|  | *Ecionemia alata* | 5/7 | 0.005457 | 0.000386 |
|  | *Erylus formosus* | 6/9 | 0.003677 | 0.000736 |
|  | *Geodia barretti* | 111/117 | < 0.000001 | < 0.000001 |
|  | *Ircinia oros* | 61/89 | < 0.000001 | < 0.000001 |
|  | *Ircinia strobilina* | 16/31 | 0.000135 | 0.000082 |
|  | *Petrosia ficiformis* | 55/101 | < 0.000001 | < 0.000001 |
|  | *Placospongia intermedia* | 4/7 | 0.037441 | 0.008065 |
|  | Plakortis simplex | 4/4 | 0.001832 | 0.000099 |
|  | *Pseudoceratina sp.* | 4/6 | 0.019165 | 0.01048 |
|  | *Rhopaloeides odorabile* | 62/62 | < 0.000001 | < 0.000001 |
|  | *Sarcotragus spinosulus* | 6/13 | 0.035057 | 0.007463 |
|  | Spheciospongia vagabunda | 4/4 | 0.001832 | 0.000259 |
|  | *Spirastrella cunctatrix* | 6/9 | 0.003677 | 0.002127 |
|  | *Spongia agaricina* | 6/11 | 0.013785 | 0.002035 |
|  | *Verongula rigida* | 5/5 | 0.000379 | 0.00001 |
|  | *Xestospongia bocatorensis* | 4/7 | 0.037441 | 0.035214 |
|  | Unspecified Porifera | 79/250 | 0.000034 | 0.000195 |
|  | Marine sediment | 18/57 | 0.035598 | 0.146646 |
