# Supplementary material for: Comparative Genomics Reveals Ecological and Evolutionary Insights into Sponge-Associated Thaumarchaeota
Source: mSystems. 2019 Aug 13;4(4):e00288-19. doi: 10.1128/mSystems.00288-19 (PMC6697440; doi:10.1128/mSystems.00288-19)
Supplement: TABLE S3 [file mSystems.00288-19-st003.docx]

| **Symbol** | **arCOG ID** | **arCOG functions** | **arCOG category** | **Cluster ID in Ca. ^U^*C. stylissum*** | **Cluster ID in Ca. ^U^*N. hexadellus*** | **Cluster ID in Ca. ^U^*N. detritiferus*** | ***Nitrosopumilus maritimus*** | **Ca. *Nitrososphaera gargensis*** | **Ca. *Nitrosopumilus koreensis*** | **Ca. Nitrososphaera evergladensis** | **Ca. Nitrosopumilus piranensis** | **Ca. Nitrocosmicus oleophilus** | **Ca. Nitrosocosmicus exaquare** | **Ca. Nitrosotenuis aquariensis** | **Ca. Nitrosotalea devanaterra** | **Ca. Nitrosopumilus sediminis** | **Ca. Nitrosopelagicus brevis** | ***Nitrososphaera viennensis*** | **Ca. Nitrosopumilus adriaticus** | **Ca. Nitrosotenuis cloacae** | **Ca. Nitrosomarinus catalina** | **Ca. ^U^*C. stylissum* ST13** | **Ca. ^U^*C. stylissum* ST14** | **Ca. ^U^*N. hexadellus* B06** | **Ca. ^U^*N. hexadellus* D6** | **Ca. ^U^*N.* *detritiferus* H8** | **Ca. ^U^*N.* *detritiferus* H13** |
| --- | --- | --- | --- | --- | --- | --- | --- | --- | --- | --- | --- | --- | --- | --- | --- | --- | --- | --- | --- | --- | --- | --- | --- | --- | --- | --- | --- |
| Functions present in Ca. UC. Stylissum, Ca. UN. hexadellus and Ca. UN. detritiferus | arCOG02926 | Plastocyanin | C | Cluster_43220\|Cluster_43024 | Cluster_1073 | Cluster_130\|Cluster_1073 | 0 | 0 | 0 | 0 | 0 | 0 | 0 | 0 | 0 | 0 | 0 | 0 | 0 | 0 | 0 | 2 | 2 | 1 | 1 | 2 | 2 |
|  | arCOG10583 | Uncharacterized membrane or secreted protein | S | Cluster_42892 | Cluster_1002 | Cluster_1002 | 0 | 0 | 0 | 0 | 0 | 0 | 0 | 0 | 0 | 0 | 0 | 0 | 0 | 0 | 0 | 1 | 1 | 1 | 1 | 1 | 1 |
|  | arCOG01739 | Signal peptidase I | U | Cluster_43101 | Cluster_1330 | Cluster_1330 | 0 | 0 | 0 | 0 | 0 | 0 | 0 | 0 | 0 | 0 | 0 | 0 | 0 | 0 | 0 | 1 | 1 | 1 | 1 | 1 | 1 |
|  | arCOG00925 | ABC transporter, ATPase, LivG | E | Cluster_43093 | Cluster_43093 | Cluster_43093 | 0 | 0 | 0 | 0 | 0 | 0 | 0 | 0 | 0 | 0 | 0 | 0 | 0 | 0 | 0 | 1 | 1 | 1 | 1 | 1 | 1 |
|  | arCOG00724 | Transcriptional regulator, PadR family | K | Cluster_43492\|Cluster_42553 | Cluster_780\|Cluster_459 | Cluster_459 | 0 | 0 | 0 | 0 | 0 | 0 | 0 | 0 | 0 | 0 | 0 | 0 | 0 | 0 | 0 | 2 | 2 | 2 | 2 | 1 | 1 |
|  | arCOG04182 | Ribosomal protein S24E | J | Cluster_42492 | Cluster_680 | Cluster_25291 | 0 | 0 | 0 | 0 | 0 | 0 | 0 | 0 | 0 | 0 | 0 | 0 | 0 | 0 | 0 | 1 | 1 | 1 | 1 | 1 | 1 |
|  | arCOG00924 | ABC transporter, ATPase, LivF | E | Cluster_43094 | Cluster_43094 | Cluster_43094 | 0 | 0 | 0 | 0 | 0 | 0 | 0 | 0 | 0 | 0 | 0 | 0 | 0 | 0 | 0 | 1 | 1 | 1 | 1 | 1 | 1 |
|  | arCOG03038 | TPR repeat-containing protein | R | Cluster_43202\|Cluster_42345\|Cluster_42315\|Cluster_42305 | Cluster_987\|Cluster_1497 | Cluster_761 | 0 | 0 | 0 | 0 | 0 | 0 | 0 | 0 | 0 | 0 | 0 | 0 | 0 | 0 | 0 | 4 | 4 | 2 | 2 | 1 | 1 |
|  | arCOG08785 | Uncharacterized protein | S | Cluster_42358 | Cluster_722 | Cluster_25040 | 0 | 0 | 0 | 0 | 0 | 0 | 0 | 0 | 0 | 0 | 0 | 0 | 0 | 0 | 0 | 1 | 1 | 1 | 1 | 1 | 1 |
|  | arCOG04094 | Ribosomal protein L24 | J | Cluster_42640 | Cluster_331 | Cluster_331 | 0 | 0 | 0 | 0 | 0 | 0 | 0 | 0 | 0 | 0 | 0 | 0 | 0 | 0 | 0 | 1 | 1 | 1 | 1 | 1 | 1 |
|  | arCOG10402 | Uncharacterized protein | S | Cluster_42371 | Cluster_487 | Cluster_25012 | 0 | 0 | 0 | 0 | 0 | 0 | 0 | 0 | 0 | 0 | 0 | 0 | 0 | 0 | 0 | 1 | 1 | 1 | 1 | 1 | 1 |
|  | arCOG03167 | Predicted ATPase | R | Cluster_42916 | Cluster_313 | Cluster_313 | 0 | 0 | 0 | 0 | 0 | 0 | 0 | 0 | 0 | 0 | 0 | 0 | 0 | 0 | 0 | 1 | 1 | 2 | 1 | 1 | 1 |
| Functions present in Ca. UC. stylissum and Ca. UN. hexadellus | arCOG08777 | Uncharacterized protein | S | Cluster_43277 | Cluster_1179 | NA | 0 | 0 | 0 | 0 | 0 | 0 | 0 | 0 | 0 | 0 | 0 | 0 | 0 | 0 | 0 | 1 | 1 | 1 | 1 | 0 | 0 |
|  | arCOG08800 | Polycystic kidney disease (PKD) domain | S | Cluster_42405 | Cluster_362\|Cluster_138 | NA | 0 | 0 | 0 | 0 | 0 | 0 | 0 | 0 | 0 | 0 | 0 | 0 | 0 | 0 | 0 | 1 | 1 | 2 | 2 | 0 | 0 |
|  | arCOG10597 | Hemocyanin family protein, binds copper ions | R | Cluster_43021 | Cluster_1402 | NA | 0 | 0 | 0 | 0 | 0 | 0 | 0 | 0 | 0 | 0 | 0 | 0 | 0 | 0 | 0 | 1 | 1 | 1 | 1 | 0 | 0 |
|  | arCOG01764 | Transcription initiation factor TFIID TATA-box-binding protein, TBP | K | Cluster_43000 | Cluster_43000 | NA | 0 | 0 | 0 | 0 | 0 | 0 | 0 | 0 | 0 | 0 | 0 | 0 | 0 | 0 | 0 | 1 | 1 | 1 | 1 | 0 | 0 |
|  | arCOG01273 | ABC transporter, permease, LivM | E | Cluster_43090 | Cluster_43090 | NA | 0 | 0 | 0 | 0 | 0 | 0 | 0 | 0 | 0 | 0 | 0 | 0 | 0 | 0 | 0 | 1 | 1 | 1 | 1 | 0 | 0 |
| Functions present in Ca. UC. stylissum and Ca. UN. detritiferus | arCOG08716 | Uncharacterized protein | S | Cluster_42548 | NA | Cluster_24072 | 0 | 0 | 0 | 0 | 0 | 0 | 0 | 0 | 0 | 0 | 0 | 0 | 0 | 0 | 0 | 1 | 1 | 0 | 0 | 1 | 1 |
|  | arCOG03416 | DNA adenine methylase, dam | L | Cluster_43445 | NA | Cluster_1345 | 0 | 0 | 0 | 0 | 0 | 0 | 0 | 0 | 0 | 0 | 0 | 0 | 0 | 0 | 0 | 1 | 1 | 0 | 0 | 1 | 1 |
|  | arCOG11516 | Uncharacterized membrane protein | S | Cluster_43173\|Cluster_43172 | NA | Cluster_24939\|Cluster_23665 | 0 | 0 | 0 | 0 | 0 | 0 | 0 | 0 | 0 | 0 | 0 | 0 | 0 | 0 | 0 | 2 | 2 | 0 | 0 | 2 | 2 |
|  | arCOG03889 | Uncharacterized protein | S | Cluster_42890\|Cluster_42829\|Cluster_42287 | NA | Cluster_24668 | 0 | 0 | 0 | 0 | 0 | 0 | 0 | 0 | 0 | 0 | 0 | 0 | 0 | 0 | 0 | 3 | 3 | 0 | 0 | 1 | 1 |
|  | arCOG01021 | ABC transporter, permease, LivK | E | Cluster_43195\|Cluster_43096\|Cluster_43095 | NA | Cluster_43195\|Cluster_23538 | 0 | 0 | 0 | 0 | 0 | 0 | 0 | 0 | 0 | 0 | 0 | 0 | 0 | 0 | 0 | 3 | 3 | 0 | 0 | 2 | 4 |
|  | arCOG08643 | Secreted protein with C-terminal PEFG domain | S | Cluster_42549 | NA | Cluster_24857 | 0 | 0 | 0 | 0 | 0 | 0 | 0 | 0 | 0 | 0 | 0 | 0 | 0 | 0 | 0 | 1 | 1 | 0 | 0 | 1 | 1 |
|  | arCOG04989 | SAM-dependent methyltransferase | Q | Cluster_43298 | NA | Cluster_43298 | 0 | 0 | 0 | 0 | 0 | 0 | 0 | 0 | 0 | 0 | 0 | 0 | 0 | 0 | 0 | 1 | 1 | 0 | 0 | 1 | 1 |
| Functions present in Ca. UN. hexadellus and Ca. UN. detritiferus | arCOG03748 | Transcriptional regulator, MarR family | K | NA | Cluster_1254 | Cluster_1254 | 0 | 0 | 0 | 0 | 0 | 0 | 0 | 0 | 0 | 0 | 0 | 0 | 0 | 0 | 0 | 0 | 0 | 1 | 1 | 1 | 1 |
|  | arCOG11967 | Uncharacterized protein | S | NA | Cluster_503 | Cluster_24211 | 0 | 0 | 0 | 0 | 0 | 0 | 0 | 0 | 0 | 0 | 0 | 0 | 0 | 0 | 0 | 0 | 0 | 1 | 1 | 1 | 1 |
|  | arCOG02966 | PBS lyase HEAT-like repeat-containing protein | S | NA | Cluster_691 | Cluster_691 | 0 | 0 | 0 | 0 | 0 | 0 | 0 | 0 | 0 | 0 | 0 | 0 | 0 | 0 | 0 | 0 | 0 | 1 | 1 | 1 | 1 |
|  | arCOG04153 | RNA polymerase III subunit C34, Lrp/AsnC family | K | NA | Cluster_1001 | Cluster_25169 | 0 | 0 | 0 | 0 | 0 | 0 | 0 | 0 | 0 | 0 | 0 | 0 | 0 | 0 | 0 | 0 | 0 | 1 | 1 | 1 | 1 |
|  | arCOG10571 | Uncharacterized protein | S | NA | Cluster_1229 | Cluster_1229 | 0 | 0 | 0 | 0 | 0 | 0 | 0 | 0 | 0 | 0 | 0 | 0 | 0 | 0 | 0 | 0 | 0 | 1 | 1 | 1 | 1 |
|  | arCOG04994 | Adamalysin (peptidase M10A) and matrixin (peptidase M12B) | E | NA | Cluster_302\|Cluster_142 | Cluster_302 | 0 | 0 | 0 | 0 | 0 | 0 | 0 | 0 | 0 | 0 | 0 | 0 | 0 | 0 | 0 | 0 | 0 | 2 | 2 | 1 | 1 |
|  | arCOG10532 | Uncharacterized protein | S | NA | Cluster_1144 | Cluster_1144 | 0 | 0 | 0 | 0 | 0 | 0 | 0 | 0 | 0 | 0 | 0 | 0 | 0 | 0 | 0 | 0 | 0 | 1 | 1 | 1 | 1 |
|  | arCOG01444 | CRISPR-associated endonuclease/helicase Cas3 | V | NA | Cluster_635 | Cluster_635 | 0 | 0 | 0 | 0 | 0 | 0 | 0 | 0 | 0 | 0 | 0 | 0 | 0 | 0 | 0 | 0 | 0 | 1 | 1 | 1 | 1 |
|  | arCOG04098 | Ribosomal protein L22 | J | NA | Cluster_337 | Cluster_337 | 0 | 0 | 0 | 0 | 0 | 0 | 0 | 0 | 0 | 0 | 0 | 0 | 0 | 0 | 0 | 0 | 0 | 1 | 1 | 1 | 1 |
|  | arCOG08185 | Uncharacterized protein | S | NA | Cluster_1152 | Cluster_1152 | 0 | 0 | 0 | 0 | 0 | 0 | 0 | 0 | 0 | 0 | 0 | 0 | 0 | 0 | 0 | 0 | 0 | 1 | 1 | 1 | 1 |
|  | arCOG01042 | Exosome subunit, RNA binding protein with dsRBD fold | J | NA | Cluster_171 | Cluster_171 | 0 | 0 | 0 | 0 | 0 | 0 | 0 | 0 | 0 | 0 | 0 | 0 | 0 | 0 | 0 | 0 | 0 | 1 | 1 | 1 | 1 |
| Functions present in Ca. UC. Stylissum | arCOG03561 | Secreted protein with beta-propeller repeat domain | R | Cluster_42955\|Cluster_42312 | NA | NA | 0 | 0 | 0 | 0 | 0 | 0 | 0 | 0 | 0 | 0 | 0 | 0 | 0 | 0 | 0 | 2 | 4 | 0 | 0 | 0 | 0 |
|  | arCOG08721 | Uncharacterized protein | S | Cluster_43526 | NA | NA | 0 | 0 | 0 | 0 | 0 | 0 | 0 | 0 | 0 | 0 | 0 | 0 | 0 | 0 | 0 | 1 | 1 | 0 | 0 | 0 | 0 |
|  | arCOG02868 | Protein-disulfide isomerase | O | Cluster_43524 | NA | NA | 0 | 0 | 0 | 0 | 0 | 0 | 0 | 0 | 0 | 0 | 0 | 0 | 0 | 0 | 0 | 1 | 1 | 0 | 0 | 0 | 0 |
|  | arCOG04157 | DNA (cytosine-5)-methyltransferase 1, dcm | L | Cluster_43520\|Cluster_43327 | NA | NA | 0 | 0 | 0 | 0 | 0 | 0 | 0 | 0 | 0 | 0 | 0 | 0 | 0 | 0 | 0 | 2 | 2 | 0 | 0 | 0 | 0 |
|  | arCOG10552 | Uncharacterized protein | S | Cluster_43518 | NA | NA | 0 | 0 | 0 | 0 | 0 | 0 | 0 | 0 | 0 | 0 | 0 | 0 | 0 | 0 | 0 | 1 | 1 | 0 | 0 | 0 | 0 |
|  | arCOG11395 | Uncharacterized protein | S | Cluster_43516 | NA | NA | 0 | 0 | 0 | 0 | 0 | 0 | 0 | 0 | 0 | 0 | 0 | 0 | 0 | 0 | 0 | 1 | 1 | 0 | 0 | 0 | 0 |
|  | arCOG02625 | Predicted metal-dependent hydrolase | R | Cluster_43511 | NA | NA | 0 | 0 | 0 | 0 | 0 | 0 | 0 | 0 | 0 | 0 | 0 | 0 | 0 | 0 | 0 | 1 | 1 | 0 | 0 | 0 | 0 |
|  | arCOG01259 | Short-chain alcohol dehydrogenase | I | Cluster_43497 | NA | NA | 0 | 0 | 0 | 0 | 0 | 0 | 0 | 0 | 0 | 0 | 0 | 0 | 0 | 0 | 0 | 1 | 1 | 0 | 0 | 0 | 0 |
|  | arCOG06586 | Uncharacterized protein | S | Cluster_43495\|Cluster_43092 | NA | NA | 0 | 0 | 0 | 0 | 0 | 0 | 0 | 0 | 0 | 0 | 0 | 0 | 0 | 0 | 0 | 2 | 2 | 0 | 0 | 0 | 0 |
|  | arCOG10518 | Uncharacterized membrane protein | S | Cluster_43490 | NA | NA | 0 | 0 | 0 | 0 | 0 | 0 | 0 | 0 | 0 | 0 | 0 | 0 | 0 | 0 | 0 | 1 | 1 | 0 | 0 | 0 | 0 |
|  | arCOG08028 | DNA repair and recombination protein RadB homolog | L | Cluster_43483 | NA | NA | 0 | 0 | 0 | 0 | 0 | 0 | 0 | 0 | 0 | 0 | 0 | 0 | 0 | 0 | 0 | 1 | 1 | 0 | 0 | 0 | 0 |
|  | arCOG00115 | Adenine-specific DNA methylase, YhdJ | L | Cluster_43479\|Cluster_42475 | NA | NA | 0 | 0 | 0 | 0 | 0 | 0 | 0 | 0 | 0 | 0 | 0 | 0 | 0 | 0 | 0 | 2 | 2 | 0 | 0 | 0 | 0 |
|  | arCOG08752 | Uncharacterized protein | S | Cluster_43456 | NA | NA | 0 | 0 | 0 | 0 | 0 | 0 | 0 | 0 | 0 | 0 | 0 | 0 | 0 | 0 | 0 | 1 | 1 | 0 | 0 | 0 | 0 |
|  | arCOG01044 | Siroheme synthase (precorrin-2 oxidase/ferrochelatase domain) | H | Cluster_43432 | NA | NA | 0 | 0 | 0 | 0 | 0 | 0 | 0 | 0 | 0 | 0 | 0 | 0 | 0 | 0 | 0 | 1 | 1 | 0 | 0 | 0 | 0 |
|  | arCOG03377 | Serine protease | E | Cluster_43410\|Cluster_43103\|Cluster_42277 | NA | NA | 0 | 0 | 0 | 0 | 0 | 0 | 0 | 0 | 0 | 0 | 0 | 0 | 0 | 0 | 0 | 3 | 3 | 0 | 0 | 0 | 0 |
|  | arCOG03788 | Late embryogenesis abundant (LEA14)-like protein | V | Cluster_43409 | NA | NA | 0 | 0 | 0 | 0 | 0 | 0 | 0 | 0 | 0 | 0 | 0 | 0 | 0 | 0 | 0 | 1 | 1 | 0 | 0 | 0 | 0 |
|  | arCOG11288 | Uncharacterized membrane protein | S | Cluster_43375 | NA | NA | 0 | 0 | 0 | 0 | 0 | 0 | 0 | 0 | 0 | 0 | 0 | 0 | 0 | 0 | 0 | 1 | 1 | 0 | 0 | 0 | 0 |
|  | arCOG07546 | Zincin superfamily protease | E | Cluster_43239 | NA | NA | 0 | 0 | 0 | 0 | 0 | 0 | 0 | 0 | 0 | 0 | 0 | 0 | 0 | 0 | 0 | 1 | 1 | 0 | 0 | 0 | 0 |
|  | arCOG00551 | DNA replication initiation complex subunit, GINS15 family | L | Cluster_43227 | NA | NA | 0 | 0 | 0 | 0 | 0 | 0 | 0 | 0 | 0 | 0 | 0 | 0 | 0 | 0 | 0 | 1 | 1 | 0 | 0 | 0 | 0 |
|  | arCOG08809 | Uncharacterized membrane protein | S | Cluster_43226 | NA | NA | 0 | 0 | 0 | 0 | 0 | 0 | 0 | 0 | 0 | 0 | 0 | 0 | 0 | 0 | 0 | 1 | 1 | 0 | 0 | 0 | 0 |
|  | arCOG00650 | Precorrin-6B methylase, cbiE | H | Cluster_43221 | NA | NA | 0 | 0 | 0 | 0 | 0 | 0 | 0 | 0 | 0 | 0 | 0 | 0 | 0 | 0 | 0 | 1 | 1 | 0 | 0 | 0 | 0 |
|  | arCOG12670 | Uncharacterized protein | S | Cluster_43218 | NA | NA | 0 | 0 | 0 | 0 | 0 | 0 | 0 | 0 | 0 | 0 | 0 | 0 | 0 | 0 | 0 | 1 | 1 | 0 | 0 | 0 | 0 |
|  | arCOG01403 | Glycosyltransferase | M | Cluster_43189 | NA | NA | 0 | 0 | 0 | 0 | 0 | 0 | 0 | 0 | 0 | 0 | 0 | 0 | 0 | 0 | 0 | 1 | 1 | 0 | 0 | 0 | 0 |
|  | arCOG02216 | MATE family membrane protein | R | Cluster_43188 | NA | NA | 0 | 0 | 0 | 0 | 0 | 0 | 0 | 0 | 0 | 0 | 0 | 0 | 0 | 0 | 0 | 1 | 1 | 0 | 0 | 0 | 0 |
|  | arCOG03441 | Uncharacterized protein | S | Cluster_43170 | NA | NA | 0 | 0 | 0 | 0 | 0 | 0 | 0 | 0 | 0 | 0 | 0 | 0 | 0 | 0 | 0 | 1 | 1 | 0 | 0 | 0 | 0 |
|  | arCOG02846 | DnaJ-class molecular chaperone | O | Cluster_43166 | NA | NA | 0 | 0 | 0 | 0 | 0 | 0 | 0 | 0 | 0 | 0 | 0 | 0 | 0 | 0 | 0 | 1 | 1 | 0 | 0 | 0 | 0 |
|  | arCOG03444 | Cupin domain containing protein | S | Cluster_43161 | NA | NA | 0 | 0 | 0 | 0 | 0 | 0 | 0 | 0 | 0 | 0 | 0 | 0 | 0 | 0 | 0 | 1 | 1 | 0 | 0 | 0 | 0 |
|  | arCOG07440 | Predicted metal-binding protein | S | Cluster_43145 | NA | NA | 0 | 0 | 0 | 0 | 0 | 0 | 0 | 0 | 0 | 0 | 0 | 0 | 0 | 0 | 0 | 1 | 1 | 0 | 0 | 0 | 0 |
|  | arCOG00507 | Metal-dependent hydrolase of the beta-lactamase superfamily II | R | Cluster_43143 | NA | NA | 0 | 0 | 0 | 0 | 0 | 0 | 0 | 0 | 0 | 0 | 0 | 0 | 0 | 0 | 0 | 1 | 1 | 0 | 0 | 0 | 0 |
|  | arCOG03233 | Predicted ATPase | R | Cluster_43137\|Cluster_42874\|Cluster_42867\|Cluster_42807\|Cluster_42280 | NA | NA | 0 | 0 | 0 | 0 | 0 | 0 | 0 | 0 | 0 | 0 | 0 | 0 | 0 | 0 | 0 | 5 | 5 | 0 | 0 | 0 | 0 |
|  | arCOG08038 | Putative membrane-associated protease inhibitor, contains alpha-2-macroglobulin-like domain | O | Cluster_43105 | NA | NA | 0 | 0 | 0 | 0 | 0 | 0 | 0 | 0 | 0 | 0 | 0 | 0 | 0 | 0 | 0 | 1 | 1 | 0 | 0 | 0 | 0 |
|  | arCOG01817 | ATPase involved in archaellum/pili biosynthesis | N | Cluster_43010 | NA | NA | 0 | 0 | 0 | 0 | 0 | 0 | 0 | 0 | 0 | 0 | 0 | 0 | 0 | 0 | 0 | 1 | 1 | 0 | 0 | 0 | 0 |
|  | arCOG00280 | HerA helicase | L | Cluster_43009 | NA | NA | 0 | 0 | 0 | 0 | 0 | 0 | 0 | 0 | 0 | 0 | 0 | 0 | 0 | 0 | 0 | 1 | 1 | 0 | 0 | 0 | 0 |
|  | arCOG03042 | TPR repeat-containing protein | R | Cluster_42997 | NA | NA | 0 | 0 | 0 | 0 | 0 | 0 | 0 | 0 | 0 | 0 | 0 | 0 | 0 | 0 | 0 | 1 | 1 | 0 | 0 | 0 | 0 |
|  | arCOG03681 | Uncharacterized protein | S | Cluster_42990 | NA | NA | 0 | 0 | 0 | 0 | 0 | 0 | 0 | 0 | 0 | 0 | 0 | 0 | 0 | 0 | 0 | 1 | 1 | 0 | 0 | 0 | 0 |
|  | arCOG09740 | Uncharacterized protein | S | Cluster_42973 | NA | NA | 0 | 0 | 0 | 0 | 0 | 0 | 0 | 0 | 0 | 0 | 0 | 0 | 0 | 0 | 0 | 1 | 1 | 0 | 0 | 0 | 0 |
|  | arCOG07571 | Uncharacterized membrane protein | S | Cluster_42964 | NA | NA | 0 | 0 | 0 | 0 | 0 | 0 | 0 | 0 | 0 | 0 | 0 | 0 | 0 | 0 | 0 | 1 | 1 | 0 | 0 | 0 | 0 |
|  | arCOG08637 | Uncharacterized protein | S | Cluster_42933 | NA | NA | 0 | 0 | 0 | 0 | 0 | 0 | 0 | 0 | 0 | 0 | 0 | 0 | 0 | 0 | 0 | 1 | 1 | 0 | 0 | 0 | 0 |
|  | arCOG08741 | Metal-dependent membrane protease, CAAX family | E | Cluster_42926 | NA | NA | 0 | 0 | 0 | 0 | 0 | 0 | 0 | 0 | 0 | 0 | 0 | 0 | 0 | 0 | 0 | 1 | 1 | 0 | 0 | 0 | 0 |
|  | arCOG01981 | Transcription initiation factor TFIIB | K | Cluster_42925\|Cluster_42381 | NA | NA | 0 | 0 | 0 | 0 | 0 | 0 | 0 | 0 | 0 | 0 | 0 | 0 | 0 | 0 | 0 | 2 | 2 | 0 | 0 | 0 | 0 |
|  | arCOG08642 | Uncharacterized protein | S | Cluster_42902 | NA | NA | 0 | 0 | 0 | 0 | 0 | 0 | 0 | 0 | 0 | 0 | 0 | 0 | 0 | 0 | 0 | 1 | 1 | 0 | 0 | 0 | 0 |
|  | arCOG08827 | Uncharacterized membrane protein | S | Cluster_42856 | NA | NA | 0 | 0 | 0 | 0 | 0 | 0 | 0 | 0 | 0 | 0 | 0 | 0 | 0 | 0 | 0 | 1 | 1 | 0 | 0 | 0 | 0 |
|  | arCOG08685 | Uncharacterized protein | S | Cluster_42849 | NA | NA | 0 | 0 | 0 | 0 | 0 | 0 | 0 | 0 | 0 | 0 | 0 | 0 | 0 | 0 | 0 | 1 | 1 | 0 | 0 | 0 | 0 |
|  | arCOG08634 | Uncharacterized protein | S | Cluster_42840 | NA | NA | 0 | 0 | 0 | 0 | 0 | 0 | 0 | 0 | 0 | 0 | 0 | 0 | 0 | 0 | 0 | 1 | 1 | 0 | 0 | 0 | 0 |
|  | arCOG04240 | Ribosomal protein S11 | J | Cluster_42821 | NA | NA | 0 | 0 | 0 | 0 | 0 | 0 | 0 | 0 | 0 | 0 | 0 | 0 | 0 | 0 | 0 | 1 | 1 | 0 | 0 | 0 | 0 |
|  | arCOG05529 | DNA-binding TFAR19-related protein, PDSD5 family | R | Cluster_42819 | NA | NA | 0 | 0 | 0 | 0 | 0 | 0 | 0 | 0 | 0 | 0 | 0 | 0 | 0 | 0 | 0 | 1 | 1 | 0 | 0 | 0 | 0 |
|  | arCOG08691 | Uncharacterized protein | S | Cluster_42816 | NA | NA | 0 | 0 | 0 | 0 | 0 | 0 | 0 | 0 | 0 | 0 | 0 | 0 | 0 | 0 | 0 | 1 | 1 | 0 | 0 | 0 | 0 |
|  | arCOG08814 | Uncharacterized protein | S | Cluster_42785 | NA | NA | 0 | 0 | 0 | 0 | 0 | 0 | 0 | 0 | 0 | 0 | 0 | 0 | 0 | 0 | 0 | 1 | 1 | 0 | 0 | 0 | 0 |
|  | arCOG04772 | Molecular chaperone, GrpE (heat shock protein) | O | Cluster_42777 | NA | NA | 0 | 0 | 0 | 0 | 0 | 0 | 0 | 0 | 0 | 0 | 0 | 0 | 0 | 0 | 0 | 1 | 1 | 0 | 0 | 0 | 0 |
|  | arCOG02726 | tRNA(Asp/Glu) amidotransferase C subunit, GATC | J | Cluster_42773 | NA | NA | 0 | 0 | 0 | 0 | 0 | 0 | 0 | 0 | 0 | 0 | 0 | 0 | 0 | 0 | 0 | 1 | 1 | 0 | 0 | 0 | 0 |
|  | arCOG00255 | Prephenate dehydratase | E | Cluster_42764 | NA | NA | 0 | 0 | 0 | 0 | 0 | 0 | 0 | 0 | 0 | 0 | 0 | 0 | 0 | 0 | 0 | 1 | 1 | 0 | 0 | 0 | 0 |
|  | arCOG02777 | Restriction endonuclease | V | Cluster_42757 | NA | NA | 0 | 0 | 0 | 0 | 0 | 0 | 0 | 0 | 0 | 0 | 0 | 0 | 0 | 0 | 0 | 1 | 1 | 0 | 0 | 0 | 0 |
|  | arCOG04345 | RNase P subunit RPR2 | J | Cluster_42726 | NA | NA | 0 | 0 | 0 | 0 | 0 | 0 | 0 | 0 | 0 | 0 | 0 | 0 | 0 | 0 | 0 | 1 | 1 | 0 | 0 | 0 | 0 |
|  | arCOG08732 | Uncharacterized protein | S | Cluster_42722 | NA | NA | 0 | 0 | 0 | 0 | 0 | 0 | 0 | 0 | 0 | 0 | 0 | 0 | 0 | 0 | 0 | 1 | 1 | 0 | 0 | 0 | 0 |
|  | arCOG01336 | Uncharacterized protein | S | Cluster_42713 | NA | NA | 0 | 0 | 0 | 0 | 0 | 0 | 0 | 0 | 0 | 0 | 0 | 0 | 0 | 0 | 0 | 1 | 1 | 0 | 0 | 0 | 0 |
|  | arCOG00038 | tRNA S(4)U 4-thiouridine synthase | J | Cluster_42704 | NA | NA | 0 | 0 | 0 | 0 | 0 | 0 | 0 | 0 | 0 | 0 | 0 | 0 | 0 | 0 | 0 | 1 | 1 | 0 | 0 | 0 | 0 |
|  | arCOG10409 | Uncharacterized protein | S | Cluster_42670 | NA | NA | 0 | 0 | 0 | 0 | 0 | 0 | 0 | 0 | 0 | 0 | 0 | 0 | 0 | 0 | 0 | 1 | 1 | 0 | 0 | 0 | 0 |
|  | arCOG08707 | Uncharacterized protein associated with Vps4/ESCRTIII system | D | Cluster_42664 | NA | NA | 0 | 0 | 0 | 0 | 0 | 0 | 0 | 0 | 0 | 0 | 0 | 0 | 0 | 0 | 0 | 1 | 1 | 0 | 0 | 0 | 0 |
|  | arCOG04097 | Ribosomal protein S3 | J | Cluster_42645 | NA | NA | 0 | 0 | 0 | 0 | 0 | 0 | 0 | 0 | 0 | 0 | 0 | 0 | 0 | 0 | 0 | 1 | 1 | 0 | 0 | 0 | 0 |
|  | arCOG08677 | Zn-ribbon domain containing protein | S | Cluster_42613 | NA | NA | 0 | 0 | 0 | 0 | 0 | 0 | 0 | 0 | 0 | 0 | 0 | 0 | 0 | 0 | 0 | 1 | 1 | 0 | 0 | 0 | 0 |
|  | arCOG03644 | Uncharacterized protein | S | Cluster_42589 | NA | NA | 0 | 0 | 0 | 0 | 0 | 0 | 0 | 0 | 0 | 0 | 0 | 0 | 0 | 0 | 0 | 1 | 1 | 0 | 0 | 0 | 0 |
|  | arCOG00347 | Archaeal enzyme of ATP-grasp superfamily | R | Cluster_42585 | NA | NA | 0 | 0 | 0 | 0 | 0 | 0 | 0 | 0 | 0 | 0 | 0 | 0 | 0 | 0 | 0 | 1 | 1 | 0 | 0 | 0 | 0 |
|  | arCOG10515 | Uncharacterized protein | S | Cluster_42579 | NA | NA | 0 | 0 | 0 | 0 | 0 | 0 | 0 | 0 | 0 | 0 | 0 | 0 | 0 | 0 | 0 | 1 | 1 | 0 | 0 | 0 | 0 |
|  | arCOG04231 | Uncharacterized protein involved in tolerance to divalent cations | P | Cluster_42576 | NA | NA | 0 | 0 | 0 | 0 | 0 | 0 | 0 | 0 | 0 | 0 | 0 | 0 | 0 | 0 | 0 | 1 | 1 | 0 | 0 | 0 | 0 |
|  | arCOG04414 | Subunit of KEOPS complex (Cgi121BUD32KAE1) | J | Cluster_42564 | NA | NA | 0 | 0 | 0 | 0 | 0 | 0 | 0 | 0 | 0 | 0 | 0 | 0 | 0 | 0 | 0 | 1 | 1 | 0 | 0 | 0 | 0 |
|  | arCOG00397 | DNA repair exonuclease, SbcD | L | Cluster_42562 | NA | NA | 0 | 0 | 0 | 0 | 0 | 0 | 0 | 0 | 0 | 0 | 0 | 0 | 0 | 0 | 0 | 1 | 1 | 0 | 0 | 0 | 0 |
|  | arCOG10525 | Uncharacterized protein | S | Cluster_42558 | NA | NA | 0 | 0 | 0 | 0 | 0 | 0 | 0 | 0 | 0 | 0 | 0 | 0 | 0 | 0 | 0 | 1 | 1 | 0 | 0 | 0 | 0 |
|  | arCOG00786 | CRISPR-associated endonuclease Cas4 | V | Cluster_42557 | NA | NA | 0 | 0 | 0 | 0 | 0 | 0 | 0 | 0 | 0 | 0 | 0 | 0 | 0 | 0 | 0 | 1 | 1 | 0 | 0 | 0 | 0 |
|  | arCOG00475 | 5,10-methylenetetrahydrofolate reductase | E | Cluster_42555 | NA | NA | 0 | 0 | 0 | 0 | 0 | 0 | 0 | 0 | 0 | 0 | 0 | 0 | 0 | 0 | 0 | 1 | 1 | 0 | 0 | 0 | 0 |
|  | arCOG03110 | Predicted transciptional regulator, contains Fic and DeoR-like domains | K | Cluster_42531 | NA | NA | 0 | 0 | 0 | 0 | 0 | 0 | 0 | 0 | 0 | 0 | 0 | 0 | 0 | 0 | 0 | 1 | 1 | 0 | 0 | 0 | 0 |
|  | arCOG03192 | Endonuclease YncB, thermonuclease family | L | Cluster_42490 | NA | NA | 0 | 0 | 0 | 0 | 0 | 0 | 0 | 0 | 0 | 0 | 0 | 0 | 0 | 0 | 0 | 1 | 1 | 0 | 0 | 0 | 0 |
|  | arCOG08713 | PasT of RatAB toxin-antitoxin module | V | Cluster_42483 | NA | NA | 0 | 0 | 0 | 0 | 0 | 0 | 0 | 0 | 0 | 0 | 0 | 0 | 0 | 0 | 0 | 1 | 1 | 0 | 0 | 0 | 0 |
|  | arCOG10579 | Uncharacterized protein | S | Cluster_42399 | NA | NA | 0 | 0 | 0 | 0 | 0 | 0 | 0 | 0 | 0 | 0 | 0 | 0 | 0 | 0 | 0 | 1 | 1 | 0 | 0 | 0 | 0 |
|  | arCOG01471 | Hemerythrin HHE cation binding domain containing protein | R | Cluster_42379 | NA | NA | 0 | 0 | 0 | 0 | 0 | 0 | 0 | 0 | 0 | 0 | 0 | 0 | 0 | 0 | 0 | 1 | 1 | 0 | 0 | 0 | 0 |
|  | arCOG10553 | Uncharacterized protein | S | Cluster_42375 | NA | NA | 0 | 0 | 0 | 0 | 0 | 0 | 0 | 0 | 0 | 0 | 0 | 0 | 0 | 0 | 0 | 1 | 1 | 0 | 0 | 0 | 0 |
|  | arCOG05195 | TPR repeat-containing protein | R | Cluster_42272 | NA | NA | 0 | 0 | 0 | 0 | 0 | 0 | 0 | 0 | 0 | 0 | 0 | 0 | 0 | 0 | 0 | 1 | 1 | 0 | 0 | 0 | 0 |
| Functions present in Ca. UN. hexadellus | arCOG01917 | Zn-ribbon domain containing protein | S | NA | Cluster_991 | NA | 0 | 0 | 0 | 0 | 0 | 0 | 0 | 0 | 0 | 0 | 0 | 0 | 0 | 0 | 0 | 0 | 0 | 1 | 1 | 0 | 0 |
|  | arCOG06735 | Uncharacterized protein | S | NA | Cluster_990 | NA | 0 | 0 | 0 | 0 | 0 | 0 | 0 | 0 | 0 | 0 | 0 | 0 | 0 | 0 | 0 | 0 | 0 | 1 | 1 | 0 | 0 |
|  | arCOG05491 | Uncharacterized protein | S | NA | Cluster_961 | NA | 0 | 0 | 0 | 0 | 0 | 0 | 0 | 0 | 0 | 0 | 0 | 0 | 0 | 0 | 0 | 0 | 0 | 1 | 1 | 0 | 0 |
|  | arCOG08780 | Predicted transcriptional regulator, contains HTH domain | K | NA | Cluster_93 | NA | 0 | 0 | 0 | 0 | 0 | 0 | 0 | 0 | 0 | 0 | 0 | 0 | 0 | 0 | 0 | 0 | 0 | 1 | 1 | 0 | 0 |
|  | arCOG11528 | Uncharacterized membrane protein | S | NA | Cluster_895 | NA | 0 | 0 | 0 | 0 | 0 | 0 | 0 | 0 | 0 | 0 | 0 | 0 | 0 | 0 | 0 | 0 | 0 | 1 | 1 | 0 | 0 |
|  | arCOG04971 | Acetyltransferase (GNAT) family | K | NA | Cluster_828 | NA | 0 | 0 | 0 | 0 | 0 | 0 | 0 | 0 | 0 | 0 | 0 | 0 | 0 | 0 | 0 | 0 | 0 | 1 | 1 | 0 | 0 |
|  | arCOG04290 | PIN-domain and Zn ribbon | R | NA | Cluster_786\|Cluster_1043 | NA | 0 | 0 | 0 | 0 | 0 | 0 | 0 | 0 | 0 | 0 | 0 | 0 | 0 | 0 | 0 | 0 | 0 | 2 | 2 | 0 | 0 |
|  | arCOG02562 | Beta-propeller repeat containing protein | R | NA | Cluster_785 | NA | 0 | 0 | 0 | 0 | 0 | 0 | 0 | 0 | 0 | 0 | 0 | 0 | 0 | 0 | 0 | 0 | 0 | 1 | 1 | 0 | 0 |
|  | arCOG01551 | NADH dehydrogenase subunit C | C | NA | Cluster_736 | NA | 0 | 0 | 0 | 0 | 0 | 0 | 0 | 0 | 0 | 0 | 0 | 0 | 0 | 0 | 0 | 0 | 0 | 1 | 1 | 0 | 0 |
|  | arCOG00663 | Nucleoside-diphosphate-sugar pyrophosphorylase involved in lipopolysaccharide biosynthesis/translation initiation factor 2B, gamma/epsilon subunit | M | NA | Cluster_502 | NA | 0 | 0 | 0 | 0 | 0 | 0 | 0 | 0 | 0 | 0 | 0 | 0 | 0 | 0 | 0 | 0 | 0 | 1 | 1 | 0 | 0 |
|  | arCOG08729 | Uncharacterized protein | S | NA | Cluster_42820 | NA | 0 | 0 | 0 | 0 | 0 | 0 | 0 | 0 | 0 | 0 | 0 | 0 | 0 | 0 | 0 | 0 | 0 | 1 | 1 | 0 | 0 |
|  | arCOG10416 | Uncharacterized protein | S | NA | Cluster_397 | NA | 0 | 0 | 0 | 0 | 0 | 0 | 0 | 0 | 0 | 0 | 0 | 0 | 0 | 0 | 0 | 0 | 0 | 1 | 1 | 0 | 0 |
|  | arCOG08778 | Alpha-2-macroglobulin-like domain (A2M) protein | S | NA | Cluster_360 | NA | 0 | 0 | 0 | 0 | 0 | 0 | 0 | 0 | 0 | 0 | 0 | 0 | 0 | 0 | 0 | 0 | 0 | 1 | 1 | 0 | 0 |
|  | arCOG08092 | Predicted membrane protein, DoxD family | S | NA | Cluster_250 | NA | 0 | 0 | 0 | 0 | 0 | 0 | 0 | 0 | 0 | 0 | 0 | 0 | 0 | 0 | 0 | 0 | 0 | 1 | 1 | 0 | 0 |
|  | arCOG01369 | Nucleoside-diphosphate-sugar epimerase | M | NA | Cluster_233 | NA | 0 | 0 | 0 | 0 | 0 | 0 | 0 | 0 | 0 | 0 | 0 | 0 | 0 | 0 | 0 | 0 | 0 | 1 | 1 | 0 | 0 |
|  | arCOG01487 | Deoxycytidylate deaminase | F | NA | Cluster_194 | NA | 0 | 0 | 0 | 0 | 0 | 0 | 0 | 0 | 0 | 0 | 0 | 0 | 0 | 0 | 0 | 0 | 0 | 1 | 1 | 0 | 0 |
|  | arCOG10586 | Ammonia monooxygenase subunit X | C | NA | Cluster_159 | NA | 0 | 0 | 0 | 0 | 0 | 0 | 0 | 0 | 0 | 0 | 0 | 0 | 0 | 0 | 0 | 0 | 0 | 1 | 1 | 0 | 0 |
|  | arCOG08647 | Uncharacterized protein | S | NA | Cluster_141 | NA | 0 | 0 | 0 | 0 | 0 | 0 | 0 | 0 | 0 | 0 | 0 | 0 | 0 | 0 | 0 | 0 | 0 | 1 | 1 | 0 | 0 |
|  | arCOG01269 | ATPase, permease, LivH | E | NA | Cluster_1357 | NA | 0 | 0 | 0 | 0 | 0 | 0 | 0 | 0 | 0 | 0 | 0 | 0 | 0 | 0 | 0 | 0 | 0 | 1 | 1 | 0 | 0 |
|  | arCOG02044 | Oligosaccharyltransferase membrane subunit | M | NA | Cluster_1319 | NA | 0 | 0 | 0 | 0 | 0 | 0 | 0 | 0 | 0 | 0 | 0 | 0 | 0 | 0 | 0 | 0 | 0 | 1 | 1 | 0 | 0 |
|  | arCOG01210 | Predicted nucleotidyltransferase and HTH domain | R | NA | Cluster_1216 | NA | 0 | 0 | 0 | 0 | 0 | 0 | 0 | 0 | 0 | 0 | 0 | 0 | 0 | 0 | 0 | 0 | 0 | 1 | 1 | 0 | 0 |
|  | arCOG10544 | Uncharacterized membrane protein | S | NA | Cluster_11 | NA | 0 | 0 | 0 | 0 | 0 | 0 | 0 | 0 | 0 | 0 | 0 | 0 | 0 | 0 | 0 | 0 | 0 | 1 | 1 | 0 | 0 |
|  | arCOG03898 | Restriction endonuclease, McrA/HNH family | V | NA | Cluster_1042 | NA | 0 | 0 | 0 | 0 | 0 | 0 | 0 | 0 | 0 | 0 | 0 | 0 | 0 | 0 | 0 | 0 | 0 | 1 | 1 | 0 | 0 |
| Functions present in Ca. UN. detritiferus | arCOG00367 | NurA 5'-3' nuclease | L | NA | NA | Cluster_5 | 0 | 0 | 0 | 0 | 0 | 0 | 0 | 0 | 0 | 0 | 0 | 0 | 0 | 0 | 0 | 0 | 0 | 0 | 0 | 1 | 1 |
|  | arCOG06565 | Uncharacterized protein | S | NA | NA | Cluster_26 | 0 | 0 | 0 | 0 | 0 | 0 | 0 | 0 | 0 | 0 | 0 | 0 | 0 | 0 | 0 | 0 | 0 | 0 | 0 | 1 | 1 |
|  | arCOG08768 | Uncharacterized membrane protein | S | NA | NA | Cluster_25306 | 0 | 0 | 0 | 0 | 0 | 0 | 0 | 0 | 0 | 0 | 0 | 0 | 0 | 0 | 0 | 0 | 0 | 0 | 0 | 1 | 1 |
|  | arCOG11325 | Uncharacterized protein | S | NA | NA | Cluster_25142 | 0 | 0 | 0 | 0 | 0 | 0 | 0 | 0 | 0 | 0 | 0 | 0 | 0 | 0 | 0 | 0 | 0 | 0 | 0 | 1 | 1 |
|  | arCOG05149 | Uncharacterized protein | S | NA | NA | Cluster_25028 | 0 | 0 | 0 | 0 | 0 | 0 | 0 | 0 | 0 | 0 | 0 | 0 | 0 | 0 | 0 | 0 | 0 | 0 | 0 | 1 | 1 |
|  | arCOG10596 | Uncharacterized protein | S | NA | NA | Cluster_24969 | 0 | 0 | 0 | 0 | 0 | 0 | 0 | 0 | 0 | 0 | 0 | 0 | 0 | 0 | 0 | 0 | 0 | 0 | 0 | 1 | 1 |
|  | arCOG01062 | Transcriptional regulator, contains HTH domain | K | NA | NA | Cluster_24965 | 0 | 0 | 0 | 0 | 0 | 0 | 0 | 0 | 0 | 0 | 0 | 0 | 0 | 0 | 0 | 0 | 0 | 0 | 0 | 1 | 1 |
|  | arCOG01805 | Phosphonate ABC transporter, periplasmic phosphonate-binding protein, phnD | P | NA | NA | Cluster_24870 | 0 | 0 | 0 | 0 | 0 | 0 | 0 | 0 | 0 | 0 | 0 | 0 | 0 | 0 | 0 | 0 | 0 | 0 | 0 | 1 | 1 |
|  | arCOG08711 | Zn finger protein | S | NA | NA | Cluster_24836 | 0 | 0 | 0 | 0 | 0 | 0 | 0 | 0 | 0 | 0 | 0 | 0 | 0 | 0 | 0 | 0 | 0 | 0 | 0 | 1 | 1 |
|  | arCOG10350 | Kazal-type serine protease inhibitor | E | NA | NA | Cluster_24789 | 0 | 0 | 0 | 0 | 0 | 0 | 0 | 0 | 0 | 0 | 0 | 0 | 0 | 0 | 0 | 0 | 0 | 0 | 0 | 1 | 1 |
|  | arCOG01885 | Ribosomal protein S17E | J | NA | NA | Cluster_24671 | 0 | 0 | 0 | 0 | 0 | 0 | 0 | 0 | 0 | 0 | 0 | 0 | 0 | 0 | 0 | 0 | 0 | 0 | 0 | 1 | 1 |
|  | arCOG01048 | Uncharacterized protein | S | NA | NA | Cluster_24667 | 0 | 0 | 0 | 0 | 0 | 0 | 0 | 0 | 0 | 0 | 0 | 0 | 0 | 0 | 0 | 0 | 0 | 0 | 0 | 1 | 1 |
|  | arCOG00029 | Orotate phosphoribosyltransferase, pyrE | F | NA | NA | Cluster_24664 | 0 | 0 | 0 | 0 | 0 | 0 | 0 | 0 | 0 | 0 | 0 | 0 | 0 | 0 | 0 | 0 | 0 | 0 | 0 | 1 | 1 |
|  | arCOG10414 | Uncharacterized protein | S | NA | NA | Cluster_24396 | 0 | 0 | 0 | 0 | 0 | 0 | 0 | 0 | 0 | 0 | 0 | 0 | 0 | 0 | 0 | 0 | 0 | 0 | 0 | 1 | 1 |
|  | arCOG10564 | Uncharacterized protein | S | NA | NA | Cluster_24264 | 0 | 0 | 0 | 0 | 0 | 0 | 0 | 0 | 0 | 0 | 0 | 0 | 0 | 0 | 0 | 0 | 0 | 0 | 0 | 1 | 1 |
|  | arCOG08662 | Uncharacterized conserved metal-binding protein | S | NA | NA | Cluster_24248 | 0 | 0 | 0 | 0 | 0 | 0 | 0 | 0 | 0 | 0 | 0 | 0 | 0 | 0 | 0 | 0 | 0 | 0 | 0 | 1 | 1 |
|  | arCOG06831 | Death-on-curing (Doc) protein | V | NA | NA | Cluster_24047 | 0 | 0 | 0 | 0 | 0 | 0 | 0 | 0 | 0 | 0 | 0 | 0 | 0 | 0 | 0 | 0 | 0 | 0 | 0 | 1 | 1 |
|  | arCOG03669 | Subtilase family protease | O | NA | NA | Cluster_22817 | 0 | 0 | 0 | 0 | 0 | 0 | 0 | 0 | 0 | 0 | 0 | 0 | 0 | 0 | 0 | 0 | 0 | 0 | 0 | 1 | 1 |
|  | arCOG03061 | Actin-like ATPase involved in cell morphogenesis | D | NA | NA | Cluster_22816 | 0 | 0 | 0 | 0 | 0 | 0 | 0 | 0 | 0 | 0 | 0 | 0 | 0 | 0 | 0 | 0 | 0 | 0 | 0 | 1 | 1 |
|  | arCOG08797 | Predicted transcriptional regulators | K | NA | NA | Cluster_1256 | 0 | 0 | 0 | 0 | 0 | 0 | 0 | 0 | 0 | 0 | 0 | 0 | 0 | 0 | 0 | 0 | 0 | 0 | 0 | 1 | 1 |
|  | arCOG08728 | Uncharacterized protein | S | NA | NA | Cluster_1087 | 0 | 0 | 0 | 0 | 0 | 0 | 0 | 0 | 0 | 0 | 0 | 0 | 0 | 0 | 0 | 0 | 0 | 0 | 0 | 1 | 1 |
| Joint function in all FL genomes | arCOG00773 | Acyl-CoA_hydrolase | I | Cluster_3196 | Cluster_3196 | Cluster_3196 | 1 | 1 | 1 | 1 | 1 | 1 | 1 | 1 | 1 | 1 | 1 | 1 | 1 | 1 | 1 | 0 | 0 | 0 | 0 | 0 | 0 |
|  | arCOG01471 | Hemerythrin_HHE_cation_binding_domain_containing_protein | R | Cluster_560\|Cluster_3210 | Cluster_3210 | Cluster_3210 | 2 | 1 | 1 | 1 | 1 | 1 | 1 | 1 | 1 | 1 | 1 | 1 | 1 | 1 | 1 | 0 | 0 | 0 | 0 | 0 | 0 |
|  | arCOG04397 | Ammonia_permease | P | Cluster_2119 | Cluster_2119 | Cluster_2119 | 1 | 1 | 1 | 1 | 1 | 1 | 1 | 1 | 2 | 1 | 1 | 1 | 1 | 1 | 1 | 0 | 0 | 0 | 0 | 0 | 0 |
|  | arCOG04233 | ABC-type_Fe3+-hydroxamate_transport_system,_periplasmic_component | P | Cluster_2538 | Cluster_2538 | Cluster_2538 | 1 | 1 | 1 | 1 | 1 | 1 | 1 | 1 | 1 | 1 | 1 | 1 | 1 | 1 | 1 | 0 | 0 | 0 | 0 | 0 | 0 |
|  | arCOG08731 | Uncharacterized_protein | S | Cluster_1969 | Cluster_1969 | Cluster_1969 | 1 | 1 | 1 | 1 | 1 | 1 | 1 | 1 | 1 | 1 | 1 | 1 | 1 | 1 | 1 | 0 | 0 | 0 | 0 | 0 | 0 |
|  | arCOG04425 | Protein-tyrosine-phosphatase | T | Cluster_2015 | Cluster_2015 | Cluster_2015 | 1 | 1 | 1 | 1 | 2 | 1 | 2 | 1 | 1 | 1 | 1 | 1 | 1 | 1 | 1 | 0 | 0 | 0 | 0 | 0 | 0 |
|  | arCOG01110 | Acetylornithine_deacetylase/Succinyl-diaminopimelate_desuccinylase_or_related_deacylase | E | Cluster_2233 | Cluster_2233 | Cluster_2233 | 1 | 1 | 1 | 1 | 1 | 2 | 2 | 1 | 1 | 1 | 1 | 1 | 1 | 1 | 1 | 0 | 0 | 0 | 0 | 0 | 0 |
|  | arCOG04641 | Uncharacterized_membrane_protein | S | Cluster_1894 | Cluster_1894 | Cluster_1894 | 1 | 1 | 1 | 1 | 1 | 1 | 1 | 1 | 1 | 1 | 1 | 1 | 1 | 1 | 1 | 0 | 0 | 0 | 0 | 0 | 0 |
| Functions abundant in FL when compared to Ca. UC. Stylissum and Ca. UN. hexadellus | arCOG01848 | Acetyltransferase_(isoleucine_patch_superfamily) | R | Cluster_1756 | Cluster_1756 | NA | 1 | 1 | 1 | 1 | 1 | 1 | 1 | 1 | 1 | 1 | 1 | 1 | 1 | 1 | 1 | 0 | 0 | 0 | 0 | NA | NA |
|  | arCOG01458 | NADPH:quinone_reductase_or_related_Zn-dependent_oxidoreductase | C | Cluster_199 | Cluster_199 | NA | 2 | 2 | 2 | 3 | 2 | 2 | 2 | 3 | 2 | 2 | 2 | 2 | 2 | 2 | 2 | 0 | 0 | 1 | 0 | NA | NA |
|  | arCOG00469 | ATPase_involved_in_DNA_replication_HolB,_small_subunit | L | Cluster_1750 | Cluster_1750 | NA | 1 | 1 | 1 | 1 | 1 | 1 | 1 | 1 | 1 | 1 | 1 | 1 | 1 | 1 | 1 | 0 | 0 | 0 | 0 | NA | NA |
|  | arCOG00663 | Nucleoside-diphosphate-sugar_pyrophosphorylase_involved_in_lipopolysaccharide_biosynthesis/translation_initiation_factor_2B,_gamma/epsilon_subunit | M | Cluster_2394 | Cluster_2394 | NA | 1 | 1 | 1 | 1 | 1 | 1 | 1 | 1 | 1 | 1 | 1 | 1 | 1 | 1 | 1 | 0 | 0 | 0 | 0 | NA | NA |
| Functions abundant in FL when compared to Ca. UC. Stylissum and Ca. UN. detritiferus | arCOG10524 | Uncharacterized_protein | S | Cluster_1022 | NA | Cluster_1022 | 1 | 1 | 1 | 1 | 1 | 1 | 1 | 1 | 1 | 1 | 1 | 1 | 1 | 1 | 1 | 0 | 0 | NA | NA | 0 | 0 |
|  | arCOG04214 | Uncharacterized_protein | S | Cluster_377 | NA | Cluster_377 | 1 | 1 | 1 | 1 | 1 | 1 | 1 | 1 | 1 | 1 | 1 | 1 | 1 | 1 | 1 | 0 | 0 | NA | NA | 0 | 0 |
|  | arCOG04055 | SHS2_domain_protein_implicated_in_nucleic_acid_metabolism | R | Cluster_1399 | NA | Cluster_1399 | 1 | 1 | 1 | 1 | 1 | 1 | 1 | 1 | 1 | 1 | 1 | 1 | 1 | 1 | 1 | 0 | 0 | NA | NA | 0 | 0 |
|  | arCOG04201 | Acyl-coenzyme_A_synthetase/AMP-(fatty)_acid_ligase | I | Cluster_2242 | NA | Cluster_2242 | 1 | 1 | 1 | 1 | 1 | 1 | 1 | 1 | 1 | 1 | 1 | 1 | 1 | 1 | 1 | 0 | 0 | NA | NA | 0 | 0 |
|  | arCOG08799 | Uncharacterized_protein | S | Cluster_2275 | NA | Cluster_2275 | 1 | 1 | 1 | 1 | 1 | 1 | 1 | 1 | 1 | 1 | 1 | 1 | 1 | 1 | 1 | 0 | 0 | NA | NA | 0 | 0 |
| Functions abundant in FL when compared to Ca. UN. hexadellus and Ca. UN. detritiferus | arCOG00397 | DNA_repair_exonuclease,_SbcD | L | NA | Cluster_42749 | Cluster_42749 | 1 | 1 | 1 | 1 | 1 | 1 | 1 | 1 | 1 | 1 | 1 | 1 | 1 | 1 | 1 | NA | NA | 0 | 0 | 0 | 0 |
|  | arCOG00280 | HerA_helicase | L | NA | Cluster_42750 | Cluster_42750 | 1 | 1 | 1 | 1 | 1 | 1 | 1 | 1 | 1 | 1 | 1 | 1 | 1 | 1 | 1 | NA | NA | 0 | 0 | 0 | 0 |
|  | arCOG04415 | Phosphoribosylamine-glycine_ligase | F | NA | Cluster_42472 | Cluster_42472 | 1 | 1 | 1 | 1 | 1 | 1 | 1 | 1 | 1 | 1 | 1 | 1 | 1 | 1 | 1 | NA | NA | 0 | 0 | 0 | 0 |
|  | arCOG05495 | Uncharacterized_protein,_contains_N-terminal_coiled-coil_domain | S | NA | Cluster_42755 | Cluster_42755 | 1 | 1 | 1 | 1 | 1 | 1 | 1 | 1 | 1 | 1 | 1 | 1 | 1 | 1 | 1 | NA | NA | 0 | 0 | 0 | 0 |
|  | arCOG04176 | Translation_initiation_factor_6_(eIF-6) | J | NA | Cluster_43381 | Cluster_43381 | 1 | 1 | 1 | 1 | 1 | 1 | 1 | 1 | 1 | 1 | 1 | 1 | 1 | 1 | 1 | NA | NA | 0 | 0 | 0 | 0 |
|  | arCOG01123 | Methylthioribose-1-phosphate_isomerase_(methionine_salvage_pathway),_a_paralog_of_eIF-2B_alpha_subunit | E | NA | Cluster_43085 | Cluster_43085 | 1 | 1 | 1 | 1 | 1 | 1 | 1 | 1 | 1 | 1 | 1 | 1 | 1 | 1 | 1 | NA | NA | 0 | 0 | 0 | 0 |
|  | arCOG03838 | Coenzyme_PQQ_synthesis_protein_D | H | NA | Cluster_43086 | Cluster_43086 | 1 | 1 | 1 | 1 | 1 | 1 | 1 | 1 | 1 | 1 | 1 | 1 | 1 | 1 | 1 | NA | NA | 0 | 0 | 0 | 0 |
|  | arCOG04098 | Ribosomal_protein_L22 | J | NA | Cluster_42646 | Cluster_42646 | 1 | 1 | 1 | 1 | 1 | 1 | 1 | 1 | 1 | 1 | 1 | 1 | 1 | 1 | 1 | NA | NA | 0 | 0 | 0 | 0 |
|  | arCOG00404 | Histidyl-tRNA_synthetase | J | NA | Cluster_43382 | Cluster_43382 | 1 | 1 | 1 | 1 | 1 | 1 | 1 | 1 | 1 | 1 | 1 | 1 | 1 | 1 | 1 | NA | NA | 0 | 0 | 0 | 0 |
|  | arCOG00412 | Phenylalanyl-tRNA_synthetase_beta_subunit | J | NA | Cluster_3089 | Cluster_3089 | 1 | 1 | 1 | 1 | 1 | 1 | 1 | 1 | 1 | 1 | 1 | 1 | 1 | 1 | 1 | NA | NA | 0 | 0 | 0 | 0 |
| Functions abundant in FL genomes when compared to Ca. UC. Stylissum | arCOG01033 | Shikimate_5-dehydrogenase | E | Cluster_1084 | NA | NA | 1 | 1 | 1 | 1 | 1 | 1 | 1 | 1 | 1 | 1 | 1 | 1 | 1 | 1 | 1 | 0 | 0 | NA | NA | NA | NA |
|  | arCOG01537 | NADH:ubiquinone_oxidoreductase_subunit_4_(chain_M) | C | Cluster_742 | NA | NA | 1 | 1 | 1 | 1 | 1 | 1 | 1 | 1 | 1 | 1 | 1 | 1 | 1 | 1 | 1 | 0 | 0 | NA | NA | NA | NA |
|  | arCOG00917 | Adenosylmethionine-8-amino-7-oxononanoate_aminotransferase | H | Cluster_1329 | NA | NA | 1 | 1 | 1 | 1 | 1 | 1 | 1 | 1 | 1 | 1 | 1 | 1 | 1 | 1 | 1 | 0 | 0 | NA | NA | NA | NA |
|  | arCOG01349 | Archaeal_fructose-1,6-bisphosphatase_or_related_enzyme_of_inositol_monophosphatase_family | G | Cluster_726 | NA | NA | 1 | 1 | 1 | 1 | 1 | 1 | 1 | 1 | 1 | 1 | 1 | 1 | 1 | 1 | 1 | 0 | 0 | NA | NA | NA | NA |
|  | arCOG01701 | tRNA_splicing_endonuclease | J | Cluster_1432 | NA | NA | 1 | 1 | 1 | 1 | 1 | 1 | 1 | 1 | 1 | 1 | 1 | 1 | 1 | 1 | 1 | 0 | 0 | NA | NA | NA | NA |
|  | arCOG01597 | Phosphoribosylaminoimidazole_carboxylase_(NCAIR_synthetase) | F | Cluster_497 | NA | NA | 1 | 1 | 1 | 1 | 1 | 1 | 1 | 1 | 1 | 1 | 1 | 1 | 1 | 1 | 1 | 0 | 0 | NA | NA | NA | NA |
|  | arCOG01088 | Indole-3-glycerol_phosphate_synthase | E | Cluster_71 | NA | NA | 1 | 1 | 1 | 1 | 1 | 1 | 1 | 1 | 1 | 1 | 1 | 1 | 1 | 1 | 1 | 0 | 0 | NA | NA | NA | NA |
|  | arCOG01086 | Tryptophan_synthase_alpha_chain | E | Cluster_2477 | NA | NA | 1 | 1 | 1 | 1 | 1 | 1 | 1 | 1 | 1 | 1 | 1 | 1 | 1 | 1 | 1 | 0 | 0 | NA | NA | NA | NA |
|  | arCOG01433 | Tryptophan_synthase_beta_chain | E | Cluster_72 | NA | NA | 1 | 1 | 1 | 1 | 1 | 1 | 1 | 1 | 1 | 1 | 1 | 1 | 1 | 1 | 1 | 0 | 0 | NA | NA | NA | NA |
|  | arCOG07810 | Adenylate_cyclase,_family_3 | T | Cluster_493 | NA | NA | 1 | 1 | 1 | 1 | 1 | 1 | 1 | 1 | 1 | 1 | 1 | 1 | 1 | 1 | 1 | 0 | 0 | NA | NA | NA | NA |
|  | arCOG00618 | Phosphoribosylformimino-5-aminoimidazole_carboxamide_ribonucleotide_(ProFAR)_isomerase | E | Cluster_1455 | NA | NA | 1 | 1 | 1 | 1 | 1 | 1 | 1 | 1 | 1 | 1 | 1 | 1 | 1 | 1 | 1 | 0 | 0 | NA | NA | NA | NA |
|  | arCOG00403 | Seryl-tRNA_synthetase | J | Cluster_168 | NA | NA | 1 | 1 | 1 | 1 | 1 | 1 | 1 | 1 | 1 | 1 | 1 | 1 | 1 | 1 | 1 | 0 | 0 | NA | NA | NA | NA |
|  | arCOG00639 | Phosphoribosylaminoimidazole_(AIR)_synthetase | F | Cluster_830 | NA | NA | 1 | 1 | 1 | 1 | 1 | 1 | 1 | 1 | 1 | 1 | 1 | 1 | 1 | 1 | 1 | 0 | 0 | NA | NA | NA | NA |
|  | arCOG00641 | Phosphoribosylformylglycinamidine_(FGAM)_synthase,_synthetase_domain | F | Cluster_1325 | NA | NA | 1 | 1 | 1 | 1 | 1 | 1 | 1 | 1 | 1 | 1 | 1 | 1 | 1 | 1 | 1 | 0 | 0 | NA | NA | NA | NA |
|  | arCOG00181 | ABC-type_dipeptide/oligopeptide/nickel_transport_system,_ATPase_component | E | Cluster_1218 | NA | NA | 1 | 1 | 1 | 1 | 1 | 1 | 1 | 1 | 1 | 1 | 1 | 1 | 1 | 1 | 1 | 0 | 0 | NA | NA | NA | NA |
|  | arCOG04338 | Cobalamin-5-phosphate_synthase | H | Cluster_420 | NA | NA | 1 | 1 | 1 | 1 | 1 | 1 | 1 | 1 | 1 | 1 | 1 | 1 | 1 | 1 | 1 | 0 | 0 | NA | NA | NA | NA |
|  | arCOG01718 | Asp-tRNAAsn/Glu-tRNAGln_amidotransferase_B_subunit | J | Cluster_219 | NA | NA | 1 | 1 | 1 | 1 | 1 | 1 | 1 | 1 | 1 | 1 | 1 | 1 | 1 | 1 | 1 | 0 | 0 | NA | NA | NA | NA |
|  | arCOG04097 | Ribosomal_protein_S3 | J | Cluster_336 | NA | NA | 1 | 1 | 1 | 1 | 1 | 1 | 1 | 1 | 1 | 1 | 1 | 1 | 1 | 1 | 1 | 0 | 0 | NA | NA | NA | NA |
|  | arCOG04186 | Ribosomal_protein_S3AE | J | Cluster_170 | NA | NA | 1 | 1 | 1 | 1 | 1 | 1 | 1 | 1 | 1 | 1 | 1 | 1 | 1 | 1 | 1 | 0 | 0 | NA | NA | NA | NA |
|  | arCOG04239 | Ribosomal_protein_S4_or_related_protein | J | Cluster_1837 | NA | NA | 1 | 1 | 1 | 1 | 1 | 1 | 1 | 1 | 1 | 1 | 1 | 1 | 1 | 1 | 1 | 0 | 0 | NA | NA | NA | NA |
|  | arCOG00650 | Precorrin-6B_methylase_1 | H | Cluster_576 | NA | NA | 1 | 1 | 1 | 1 | 1 | 1 | 1 | 1 | 1 | 1 | 1 | 1 | 1 | 1 | 1 | 0 | 0 | NA | NA | NA | NA |
|  | arCOG02097 | 3-dehydroquinate_dehydratase | E | Cluster_1083 | NA | NA | 1 | 1 | 1 | 1 | 1 | 1 | 1 | 1 | 1 | 1 | 1 | 1 | 1 | 1 | 1 | 0 | 0 | NA | NA | NA | NA |
|  | arCOG00288 | Nitroreductase | C | Cluster_919 | NA | NA | 1 | 1 | 1 | 1 | 1 | 1 | 1 | 1 | 1 | 1 | 1 | 1 | 1 | 1 | 1 | 0 | 0 | NA | NA | NA | NA |
|  | arCOG04228 | Peptidyl-tRNA_hydrolase | J | Cluster_235 | NA | NA | 1 | 1 | 1 | 1 | 1 | 1 | 1 | 1 | 1 | 1 | 1 | 1 | 1 | 1 | 1 | 0 | 0 | NA | NA | NA | NA |
|  | arCOG04431 | Glycerol_uptake_facilitator_or_related_permease_(Major_Intrinsic_Protein_Family) | G | Cluster_1099 | NA | NA | 1 | 1 | 1 | 1 | 1 | 1 | 2 | 1 | 1 | 1 | 1 | 1 | 1 | 1 | 1 | 0 | 0 | NA | NA | NA | NA |
|  | arCOG02007 | Prenyltransferase_family_protein_containing_thioredoxin_domain | O | Cluster_1276 | NA | NA | 1 | 1 | 1 | 1 | 1 | 1 | 1 | 1 | 1 | 1 | 1 | 1 | 1 | 1 | 1 | 0 | 0 | NA | NA | NA | NA |
|  | arCOG04227 | Predicted_CoA-binding_protein | R | Cluster_962 | NA | NA | 1 | 1 | 1 | 1 | 1 | 1 | 1 | 1 | 1 | 1 | 1 | 1 | 1 | 1 | 1 | 0 | 0 | NA | NA | NA | NA |
|  | arCOG02579 | Fe-S-cluster_containining_protein | R | Cluster_1122 | NA | NA | 1 | 1 | 1 | 1 | 1 | 1 | 1 | 1 | 1 | 1 | 1 | 1 | 1 | 1 | 1 | 0 | 0 | NA | NA | NA | NA |
|  | arCOG00084 | tRNA(Ser,Leu)_C12_N-acetylase_TAN1,_contains_THUMP_domain | J | Cluster_1293 | NA | NA | 1 | 1 | 1 | 1 | 1 | 1 | 1 | 1 | 1 | 1 | 1 | 1 | 1 | 1 | 1 | 0 | 0 | NA | NA | NA | NA |
|  | arCOG02431 | Predicted_Rossmann_fold_nucleotide-binding_protein | R | Cluster_959 | NA | NA | 1 | 1 | 1 | 1 | 1 | 1 | 1 | 1 | 1 | 1 | 1 | 1 | 1 | 1 | 1 | 0 | 0 | NA | NA | NA | NA |
|  | arCOG02640 | Uncharacterized_protein_YkaA,_distantly_related_to_PhoU,_UPF0111/DUF47_family | P | Cluster_1431 | NA | NA | 1 | 1 | 1 | 1 | 1 | 1 | 1 | 1 | 1 | 1 | 1 | 1 | 1 | 1 | 1 | 0 | 0 | NA | NA | NA | NA |
|  | arCOG00978 | tRNA(1-methyladenosine)_methyltransferase | J | Cluster_248 | NA | NA | 1 | 1 | 1 | 1 | 1 | 1 | 1 | 1 | 1 | 1 | 1 | 1 | 1 | 1 | 1 | 0 | 0 | NA | NA | NA | NA |
|  | arCOG04262 | Phosphopantothenate_synthetase | H | Cluster_902 | NA | NA | 1 | 1 | 1 | 1 | 1 | 1 | 1 | 1 | 1 | 1 | 1 | 1 | 1 | 1 | 1 | 0 | 0 | NA | NA | NA | NA |
|  | arCOG01006 | ABC-type_Mn2+/Zn2+_transport_system,_permease_component | P | Cluster_268 | NA | NA | 1 | 1 | 1 | 1 | 1 | 1 | 1 | 1 | 1 | 1 | 1 | 1 | 1 | 1 | 1 | 0 | 0 | NA | NA | NA | NA |
|  | arCOG04353 | 3-dehydroquinate_synthase | E | Cluster_1082 | NA | NA | 1 | 1 | 1 | 1 | 1 | 1 | 1 | 1 | 1 | 1 | 1 | 1 | 1 | 1 | 1 | 0 | 0 | NA | NA | NA | NA |
|  | arCOG04122 | rRNA_pseudouridine-1189_N-methylase_Emg1,_Nep1/Mra1_family | J | Cluster_631 | NA | NA | 1 | 1 | 1 | 1 | 1 | 1 | 1 | 1 | 1 | 1 | 1 | 1 | 1 | 1 | 1 | 0 | 0 | NA | NA | NA | NA |
|  | arCOG01696 | 2,3-bisphosphoglycerate-independent_phosphoglycerate_mutase | G | Cluster_368 | NA | NA | 1 | 1 | 1 | 1 | 1 | 1 | 1 | 1 | 1 | 1 | 1 | 1 | 1 | 1 | 1 | 0 | 0 | NA | NA | NA | NA |
|  | arCOG00767 | Phosphomannomutase | G | Cluster_1386 | NA | NA | 1 | 1 | 1 | 1 | 1 | 1 | 1 | 1 | 1 | 1 | 1 | 1 | 1 | 1 | 1 | 0 | 0 | NA | NA | NA | NA |
|  | arCOG04044 | 2-amino-3,7-dideoxy-D-threo-hept-6-ulosonic_acid_synthase,_DhnA-aldolase_family | E | Cluster_1081 | NA | NA | 1 | 1 | 1 | 1 | 1 | 1 | 1 | 1 | 1 | 1 | 1 | 1 | 1 | 1 | 1 | 0 | 0 | NA | NA | NA | NA |
|  | arCOG00018 | NAD(P)H-hydrate_repair_enzyme_Nnr,_NAD(P)H-hydrate_dehydratase_domain | F | Cluster_249 | NA | NA | 1 | 1 | 1 | 1 | 1 | 1 | 1 | 1 | 1 | 1 | 1 | 1 | 1 | 1 | 1 | 0 | 0 | NA | NA | NA | NA |
|  | arCOG08668 | Uncharacterized_protein | S | Cluster_40 | NA | NA | 1 | 1 | 1 | 1 | 1 | 1 | 1 | 1 | 1 | 1 | 1 | 1 | 1 | 1 | 1 | 0 | 0 | NA | NA | NA | NA |
|  | arCOG08080 | Uncharacterized_protein | S | Cluster_1275 | NA | NA | 1 | 1 | 1 | 1 | 1 | 1 | 1 | 1 | 1 | 1 | 1 | 1 | 1 | 1 | 1 | 0 | 0 | NA | NA | NA | NA |
|  | arCOG07440 | Predicted_metal-binding_protein | S | Cluster_1130 | NA | NA | 1 | 1 | 1 | 1 | 1 | 1 | 1 | 1 | 1 | 1 | 1 | 1 | 1 | 1 | 1 | 0 | 0 | NA | NA | NA | NA |
|  | arCOG10590 | Uncharacterized_protein | S | Cluster_3132 | NA | NA | 1 | 1 | 1 | 1 | 2 | 1 | 1 | 1 | 1 | 1 | 1 | 1 | 1 | 1 | 1 | 0 | 0 | NA | NA | NA | NA |
|  | arCOG01093 | Protein_distantly_related_to_bacterial_ferritins | R | Cluster_1289 | NA | NA | 1 | 2 | 1 | 1 | 1 | 1 | 1 | 1 | 1 | 1 | 1 | 1 | 1 | 1 | 1 | 0 | 0 | NA | NA | NA | NA |
|  | arCOG01213 | HAD_superfamily_hydrolase | R | Cluster_972 | NA | NA | 1 | 1 | 1 | 1 | 1 | 1 | 1 | 1 | 1 | 1 | 1 | 1 | 1 | 1 | 1 | 0 | 0 | NA | NA | NA | NA |
|  | arCOG08770 | Uncharacterized_protein | S | Cluster_875 | NA | NA | 1 | 1 | 1 | 1 | 1 | 1 | 1 | 1 | 1 | 1 | 1 | 1 | 1 | 1 | 1 | 0 | 0 | NA | NA | NA | NA |
|  | arCOG01389 | Glycosyl_transferase_family_2 | M | Cluster_841 | NA | NA | 1 | 1 | 1 | 1 | 1 | 1 | 1 | 1 | 1 | 1 | 1 | 1 | 1 | 1 | 1 | 0 | 0 | NA | NA | NA | NA |
|  | arCOG01393 | Glycosyl_transferase,_related_to_UDP-glucuronosyltransferase | G | Cluster_787 | NA | NA | 1 | 1 | 1 | 1 | 1 | 1 | 1 | 1 | 1 | 1 | 1 | 1 | 1 | 1 | 1 | 0 | 0 | NA | NA | NA | NA |
|  | arCOG00692 | Cytosine_deaminase_or_related_metal-dependent_hydrolase | F | Cluster_764 | NA | NA | 1 | 1 | 1 | 1 | 1 | 1 | 1 | 1 | 1 | 1 | 1 | 1 | 1 | 1 | 1 | 0 | 0 | NA | NA | NA | NA |
|  | arCOG01845 | Metal-sulfur_cluster_biosynthetic_enzyme | O | Cluster_699 | NA | NA | 1 | 1 | 1 | 1 | 1 | 1 | 1 | 1 | 1 | 1 | 1 | 1 | 1 | 1 | 1 | 0 | 0 | NA | NA | NA | NA |
|  | arCOG00581 | PEP_phosphonomutase_or_related_enzyme | G | Cluster_693 | NA | NA | 1 | 1 | 1 | 1 | 1 | 1 | 1 | 1 | 1 | 1 | 1 | 1 | 1 | 1 | 1 | 0 | 0 | NA | NA | NA | NA |
|  | arCOG02410 | Coenzyme_F420-dependent_N5,N10-methylene_tetrahydromethanopterin_reductase_or_related_flavin-dependent_oxidoreductase | C | Cluster_504 | NA | NA | 1 | 1 | 1 | 1 | 1 | 1 | 1 | 1 | 1 | 1 | 1 | 1 | 1 | 1 | 1 | 0 | 0 | NA | NA | NA | NA |
|  | arCOG04006 | HEAT_repeats_containing_protein | S | Cluster_2840 | NA | NA | 1 | 1 | 1 | 1 | 1 | 1 | 1 | 1 | 1 | 1 | 1 | 1 | 1 | 1 | 1 | 0 | 0 | NA | NA | NA | NA |
|  | arCOG01623 | Aryl-alcohol_dehydrogenase_related_enzyme | C | Cluster_226 | NA | NA | 1 | 1 | 1 | 1 | 1 | 1 | 1 | 1 | 1 | 1 | 1 | 1 | 1 | 1 | 1 | 0 | 0 | NA | NA | NA | NA |
|  | arCOG02900 | vWFA_domain_containing_protein | R | Cluster_174 | NA | NA | 1 | 1 | 1 | 1 | 1 | 1 | 1 | 1 | 1 | 1 | 1 | 1 | 1 | 1 | 1 | 0 | 0 | NA | NA | NA | NA |
|  | arCOG08672 | Uncharacterized_membrane_protein | S | Cluster_163 | NA | NA | 1 | 1 | 1 | 1 | 1 | 1 | 1 | 1 | 1 | 1 | 1 | 1 | 1 | 1 | 1 | 0 | 0 | NA | NA | NA | NA |
|  | arCOG01628 | DNA-binding_transcriptional_regulator,_Lrp_family | K | Cluster_1495 | NA | NA | 1 | 1 | 1 | 1 | 1 | 1 | 1 | 1 | 1 | 1 | 1 | 1 | 1 | 1 | 1 | 0 | 0 | NA | NA | NA | NA |
|  | arCOG08717 | Protein_disulfide_reductases,_TlpA_family | C | Cluster_1401 | NA | NA | 1 | 1 | 1 | 1 | 1 | 1 | 1 | 1 | 1 | 1 | 1 | 1 | 1 | 1 | 1 | 0 | 0 | NA | NA | NA | NA |
|  | arCOG03008 | Cupin_domain_containing_protein | G | Cluster_1284 | NA | NA | 1 | 1 | 1 | 1 | 1 | 1 | 1 | 1 | 1 | 1 | 1 | 1 | 1 | 1 | 1 | 0 | 0 | NA | NA | NA | NA |
|  | arCOG01564 | Selenocysteine-specific_translation_elongation_factor__or_SelB-II_domain | J | Cluster_110 | NA | NA | 1 | 1 | 1 | 1 | 1 | 1 | 1 | 1 | 1 | 1 | 1 | 1 | 1 | 1 | 1 | 0 | 0 | NA | NA | NA | NA |
|  | arCOG06353 | Probable_cobalt_transporter_subunit,_CbtA | P | Cluster_208 | NA | NA | 1 | 1 | 1 | 1 | 2 | 1 | 1 | 2 | 1 | 1 | 1 | 1 | 1 | 1 | 1 | 0 | 0 | NA | NA | NA | NA |
|  | arCOG02053 | Nucleotide-binding_protein,__UspA_family | T | Cluster_968 | NA | NA | 1 | 1 | 1 | 1 | 1 | 1 | 1 | 1 | 1 | 1 | 1 | 1 | 1 | 1 | 1 | 0 | 0 | NA | NA | NA | NA |
| Functions abundant in FL genomes when compared to Ca. UN. hexadellus | arCOG00063 | CTP_synthase_(UTP-ammonia_lyase) | F | NA | Cluster_43389 | NA | 1 | 1 | 1 | 1 | 1 | 1 | 1 | 1 | 1 | 1 | 1 | 1 | 1 | 1 | 1 | NA | NA | 0 | 0 | NA | NA |
|  | arCOG00310 | Peroxiredoxin | O | NA | Cluster_42522 | NA | 1 | 1 | 1 | 1 | 1 | 1 | 1 | 1 | 1 | 1 | 1 | 1 | 1 | 1 | 1 | NA | NA | 0 | 0 | NA | NA |
|  | arCOG00415 | RecA/RadA_recombinase | L | NA | Cluster_42701 | NA | 1 | 1 | 0 | 1 | 1 | 1 | 1 | 1 | 1 | 1 | 1 | 1 | 1 | 1 | 1 | NA | NA | 0 | 0 | NA | NA |
|  | arCOG00520 | Nitroimidazol_reductase_NimA_or_a_related_FMN-containing_flavoprotein,_pyridoxamine_5'-phosphate_oxidase_superfamily | V | NA | Cluster_42365 | NA | 1 | 1 | 1 | 1 | 1 | 1 | 1 | 1 | 1 | 1 | 1 | 1 | 1 | 1 | 1 | NA | NA | 0 | 0 | NA | NA |
|  | arCOG00891 | Mg-dependent_DNase | L | NA | Cluster_42390 | NA | 1 | 1 | 1 | 1 | 1 | 1 | 1 | 1 | 1 | 1 | 1 | 1 | 1 | 1 | 1 | NA | NA | 0 | 0 | NA | NA |
|  | arCOG02043 | Dolichol_phosphate-mannose_mannosyltransferase | M | NA | Cluster_43248 | NA | 1 | 1 | 1 | 1 | 1 | 1 | 1 | 1 | 1 | 1 | 1 | 1 | 1 | 1 | 1 | NA | NA | 0 | 0 | NA | NA |
|  | arCOG02062 | TusA-related_sulfurtransferase | P | NA | Cluster_2205 | NA | 1 | 1 | 1 | 1 | 1 | 1 | 1 | 1 | 1 | 1 | 1 | 1 | 1 | 1 | 1 | NA | NA | 0 | 0 | NA | NA |
|  | arCOG02144 | Histones_H3_and_H4 | L | NA | Cluster_42391 | NA | 1 | 1 | 1 | 1 | 1 | 1 | 1 | 1 | 1 | 1 | 1 | 1 | 1 | 1 | 1 | NA | NA | 0 | 0 | NA | NA |
|  | arCOG02926 | Plastocyanin | C | NA | Cluster_2720 | NA | 1 | 2 | 1 | 4 | 2 | 1 | 1 | 1 | 2 | 1 | 1 | 1 | 2 | 1 | 1 | NA | NA | 0 | 0 | NA | NA |
|  | arCOG04112 | Diphthamide_synthase_subunit_DPH2 | J | NA | Cluster_43417 | NA | 1 | 1 | 1 | 1 | 1 | 1 | 1 | 1 | 1 | 1 | 1 | 1 | 1 | 1 | 1 | NA | NA | 0 | 0 | NA | NA |
|  | arCOG04748 | Excinuclease_ABC_subunit_B,_helicase | L | NA | Cluster_42366 | NA | 1 | 1 | 0 | 1 | 1 | 1 | 1 | 1 | 1 | 1 | 1 | 1 | 1 | 1 | 1 | NA | NA | 0 | 0 | NA | NA |
|  | arCOG07444 | S-adenosylmethionine_synthetase | H | NA | Cluster_43257 | NA | 1 | 1 | 1 | 1 | 1 | 1 | 1 | 1 | 1 | 1 | 1 | 1 | 1 | 1 | 1 | NA | NA | 0 | 0 | NA | NA |
| Functions abundant in FL genomes when compared to Ca. UN. detritiferus | arCOG00029 | Orotate_phosphoribosyltransferase | F | NA | NA | Cluster_433 | 1 | 1 | 1 | 1 | 1 | 1 | 1 | 1 | 1 | 1 | 1 | 1 | 1 | 1 | 1 | NA | NA | NA | NA | 0 | 0 |
|  | arCOG00042 | tRNA(Ile)-lysidine_synthase_TilS/MesJ | J | NA | NA | Cluster_42578 | 1 | 1 | 1 | 1 | 1 | 1 | 1 | 1 | 1 | 1 | 1 | 1 | 1 | 1 | 1 | NA | NA | NA | NA | 0 | 0 |
|  | arCOG00291 | Ferredoxin | C | NA | NA | Cluster_43036 | 1 | 1 | 1 | 1 | 1 | 1 | 1 | 1 | 1 | 1 | 1 | 1 | 1 | 1 | 1 | NA | NA | NA | NA | 0 | 0 |
|  | arCOG00689 | Dihydroorotase_or_related_cyclic_amidohydrolase | F | NA | NA | Cluster_42335 | 1 | 1 | 1 | 1 | 1 | 1 | 1 | 1 | 1 | 1 | 1 | 1 | 1 | 1 | 1 | NA | NA | NA | NA | 0 | 0 |
|  | arCOG00933 | Radical_SAM_superfamily_enzyme | R | NA | NA | Cluster_43192 | 1 | 1 | 1 | 1 | 1 | 1 | 1 | 1 | 1 | 1 | 1 | 1 | 1 | 1 | 1 | NA | NA | NA | NA | 0 | 0 |
|  | arCOG00998 | Small_nuclear_ribonucleoprotein_(snRNP)_homolog | K | NA | NA | Cluster_43132 | 1 | 1 | 1 | 1 | 1 | 1 | 1 | 1 | 1 | 1 | 1 | 1 | 1 | 1 | 1 | NA | NA | NA | NA | 0 | 0 |
|  | arCOG01122 | Ribose_5-phosphate_isomerase | G | NA | NA | Cluster_42336 | 1 | 1 | 1 | 1 | 1 | 1 | 1 | 1 | 1 | 1 | 1 | 1 | 1 | 1 | 1 | NA | NA | NA | NA | 0 | 0 |
|  | arCOG01138 | Proteasome_lid_subunit_RPN8/RPN11,_contains_Jab1/MPN_domain_metalloenzyme_(JAMM)_motif | O | NA | NA | Cluster_42629 | 1 | 1 | 1 | 1 | 1 | 1 | 1 | 1 | 1 | 1 | 1 | 1 | 1 | 1 | 1 | NA | NA | NA | NA | 0 | 0 |
|  | arCOG01590 | Biotin_carboxylase | I | NA | NA | Cluster_42360 | 1 | 1 | 1 | 1 | 1 | 1 | 1 | 1 | 1 | 1 | 1 | 1 | 1 | 1 | 1 | NA | NA | NA | NA | 0 | 0 |
|  | arCOG02100 | Mn-dependent_transcriptional_regulator_(DtxR_family) | K | NA | NA | Cluster_43194 | 1 | 1 | 1 | 1 | 1 | 1 | 1 | 1 | 1 | 1 | 1 | 1 | 1 | 1 | 1 | NA | NA | NA | NA | 0 | 0 |
|  | arCOG02826 | Formyltetrahydrofolate_hydrolase | F | NA | NA | Cluster_43134 | 1 | 1 | 1 | 1 | 1 | 1 | 1 | 1 | 1 | 1 | 1 | 1 | 1 | 1 | 1 | NA | NA | NA | NA | 0 | 0 |
|  | arCOG04033 | Uncharacterized_protein | S | NA | NA | Cluster_43215 | 1 | 1 | 1 | 1 | 1 | 1 | 1 | 1 | 1 | 1 | 1 | 1 | 1 | 1 | 1 | NA | NA | NA | NA | 0 | 0 |
|  | arCOG04536 | Creatinine_amidohydrolase/Fe(II)-dependent_formamide_hydrolase_involved_in_riboflavin_and_F420_biosynthesis | H | NA | NA | Cluster_1383 | 1 | 1 | 1 | 1 | 1 | 1 | 1 | 1 | 1 | 1 | 1 | 1 | 1 | 1 | 1 | NA | NA | NA | NA | 0 | 0 |
