# Supplementary material for: Comparative Genomics Reveals Ecological and Evolutionary Insights into Sponge-Associated Thaumarchaeota
Source: mSystems. 2019 Aug 13;4(4):e00288-19. doi: 10.1128/mSystems.00288-19 (PMC6697440; doi:10.1128/mSystems.00288-19)
Supplement: TABLE S4 [file mSystems.00288-19-st004.docx]

| Samples | Host Species | Date yy/mm | Location | Latitude/°N | Longitude /°E |
| --- | --- | --- | --- | --- | --- |
| B06 | *Hexadella dedritifera* | 2006/Jul | Mingulay, Scotland | 56.82 | -7.37 |
| D6 | *Hexadella dedritifera* | 2005/Jul | Logachev Mounds, Rockall Bank, Irish Sea, Ireland | 55.44 | -16.08 |
| H8 | *Hexadella* cf. *dedritifera* | 2011/Sep | Petite sole Canyon, Celtic Sea, France | 48.14 | -8.80 |
| H13 | *Hexadella* cf. *dedritifera* | 2011/Oct | Logachev Mounds, Rockall Bank, Irish Sea, Ireland | 55.52 | -15.65 |
| S13 | *Stylissa flabelliformis* | 2014/Aug | Davies Reef, Great Barrier Reef, Australia | -18.83 | 147.68 |
| S14 | *Stylissa flabelliformis* | 2014/Aug | Davies Reef, Great Barrier Reef, Australia | -18.83 | 147.68 |
| S15 | *Stylissa flabelliformis* | 2014/Aug | Davies Reef, Great Barrier Reef, Australia | -18.83 | 147.68 |
